# *SDR42E1* modulates vitamin D absorption and cancer pathogenesis: insights from an *in vitro* model

**DOI:** 10.3389/fendo.2025.1585859

**Published:** 2025-07-18

**Authors:** Nagham Nafiz Hendi, Georges Nemer

**Affiliations:** ^1^ Faculty of Pharmacy, Middle East University, Amman, Jordan; ^2^ Division of Genomics and Translational Biomedicine, College of Health and Life Sciences, Hamad Bin Khalifa University, Doha, Qatar; ^3^ Department of Biochemistry and Molecular Genetics, American University of Beirut, Beirut, Lebanon

**Keywords:** vitamin D regulation, SDR42E1, endocrine disorders, genetics in endocrinology, precision medicine

## Abstract

**Introduction:**

Vitamin D is a pleiotropic hormone essential for bone health and overall physiological function. Despite its significance, vitamin D deficiency remains widespread and is often influenced by genetic factors.

**Methods:**

This study investigates the role of *SDR42E1*, a gene encoding a short-chain dehydrogenase/reductase enzyme, in vitamin D regulation and sterol metabolism. Using CRISPR/Cas9 gene-editing, we generated an *SDR42E1* knock-in model in HCT116 colorectal cells, which exhibit high endogenous *SDR42E1* expression, harboring a nonsense variant associated with vitamin D deficiency.

**Results:**

Integrated transcriptomic and proteomic analyses revealed significant dysregulation of sterol absorption and metabolism (fold change (FC) = 1.8, *P* = 0.007) and cancer-related signaling pathways (FC = −1.7, *P* = 0.02). Notably, key differentially expressed genes included upregulated *LRP1B* and *ABCC2*, alongside downregulated *WNT16* and *SLC7A5*. Proteomic profiling confirmed alterations in cell proliferation-related proteins, including reduced ALDOA expression (FC = −0.37, P = 0.0005). Functionally, *SDR42E1* deficiency reduced cell viability by 53% (P = 0.0001), an effect reversed by transient *SDR42E1* overexpression with restoring ABCC2 expression.

**Conclusion:**

These findings establish *SDR42E1* as a key modulator of vitamin D-related pathways and highlight its potential as a therapeutic target for addressing vitamin D deficiency and associated pathologies, including cancer.

## 1 Introduction

Vitamin D is a vital fat-soluble nutrient crucial for calcium and phosphorus homeostasis, bone health, and immune function. Despite the availability of dietary sources and sunlight exposure (1), deficiencies can arise due to impaired absorption and metabolism. These processes are orchestrated by a network of critical proteins, like ATP-binding cassette B member 2 (ABCB2) and solute carrier family proteins, including SLC7A5 (2, 3). In circulation, vitamin D binds to vitamin D-binding protein (VDBP) and undergoes hydroxylation in the liver by cytochrome P450 enzymes such as CYP2R1, CYP11A1, and CYP27A1, which hydroxylate vitamin D, followed by activation in the kidney by CYP27B1. The vitamin D receptor (VDR) mediates its biological effects, while CYP24A1 regulates its inactivation (4). Both VDR and CYP24A1 influence these processes, although key mechanistic gaps remain incompletely understood (5).

Short-chain Dehydrogenase/Reductase 42E member 1 (SDR42E1), a member of the short-chain dehydrogenase/reductase enzyme family plays a potentially significant role in lipid and steroid metabolism (6–8), potentially functioning as an oxidoreductase and steroid delta-isomerase (9, 10). The largest genome-wide association study (GWAS) on vitamin D deficiency (11), identified a nonsense variant (rs11542462) on chromosome 16q23 that replaces glutamine with a termination codon at position 30 (p.Q30*), producing a truncated, non-functional SDR42E1 enzyme (12–14). Interestingly, this variant correlates with elevated serum levels of 8-dehydrocholesterol (8-DHC) and 7-dehydrocholesterol (7-DHC), precursors in vitamin D synthesis (15). Lately, our *in silico* studies identified these sterols, along with vitamin D_3_ and 25-hydroxyvitamin D (25(OH)D), as potential substrates of the SDR42E1 (9); however, its precise role in vitamin D metabolism remains unclear.

Our recent research, utilizing clustered regularly interspaced short palindromic repeats/CRISPR-associated protein 9 (CRISPR/Cas9) gene-editing technology in skin keratinocytes, demonstrated that *SDR42E1* depletion disrupts the steroid biosynthesis, leading to accumulation of 7-DHC and a concomitant reduction in vitamin D levels. Additionally, *SDR42E1* mRNA showed elevated expression in intestinal epithelial cells and the analogous colorectal HCT116 cell line, underscoring its putative role in vitamin D homeostasis within the gastrointestinal tract (16). Building on these observations, this study seeks to elucidate the functional role of *SDR42E1* in regulating intestinal vitamin D absorption and sterol metabolism. To achieve this, we employed CRISPR/Cas9-mediated gene editing in HCT116 cells to introduce a nonsense variant of *SDR42E1* previously associated with vitamin D deficiency. Comprehensive transcriptomic and proteomic analyses were then performed to characterize downstream molecular alterations of SDR42E1 disruption. Through this integrative approach, we aim to advance mechanistic understanding of *SDR42E1*’s contribution to vitamin D homeostasis and to explore its broader implications in metabolic regulation and disease pathogenesis.

## 2 Materials and methods

### 2.1 Cell culture

The human colorectal carcinoma cell line, HCT116 (CCL-247, Research Resource Identifier (RRID): CVCL_029), was sourced from the American Type Culture Collection (Manassas, VA, United States). Cells were cultured in Dulbecco’s modified Eagle’s medium-high D-glucose (4.5 g/L), glutamine (2 mM), and sodium pyruvate (1 mM), and further supplemented with 10% fetal bovine serum and (1X) antibiotic-antimycotic (Thermo Fisher Gibco, United States). To maintain sterility and optimal growth conditions, the culture medium was refreshed every two days. Cells were allowed to reach 70-80% confluency before being detached using 0.25% trypsin-ethylenediaminetetraacetic acid (Sigma-Aldrich), washed with Dulbecco’s phosphate-buffered saline (DPBS, Gibco), and then centrifuged at 900 revolutions per minute (rpm) for 3 minutes. The cells were then subcultured at a 1:6 ratio. The cultures were maintained in a sterile environment at 37°C, in a 5% CO_2_ and 95% humidity atmosphere, ensuring monolayer growth. A summary of key resources for materials and reagents used is presented in **Supplementary Table S1**.

### 2.2 Plasmid construction and cloning

To assess the potential role of the SDR42E1 protein, wild-type *SDR42E1* (pcDNA3.1+/N-HA) plasmid was procured from GenScript (USA). Wild-type *SDR42E1* was transiently overexpressed using a pair of primers (forward: GGGCTACACATTCCCGTCTA, reverse: AACCATTCCACTGCTTCCTG, amplicon size: 231 base pair, primer length: 20 nucleotides). Clones were amplified in DH5α competent *Escherichia coli* (*E. coli*) and validated by Sanger sequencing and Western blotting analyses. HCT116 cells, at 40-60% confluency, were transiently transfected with the wild-type plasmids using Lipofectamine 3000 (Thermo Fisher, USA), for 24 hours per manufacturer’s instructions.

### 2.3 Creation of *SDR42E1* knock-in cells using CRISPR/Cas9

A loss-of-function p.Q30* nonsense mutation in the *SDR42E1* gene, recapitulating the variant observed in patients, was introduced into HCT116 cells using CRISPR/Cas9 technology (GenScript Biotech, https://www.genscript.com/, USA, Order ID: U864NGG050). This was achieved by inserting a G>A substitution into exon 3 using a guide RNA (gRNA) and Cas9 protein delivered via the eSpCas9-2A-GFP (PX458) plasmid (**Supplementary Figure S1**). For this process, HCT116 cells were co-transfected with a donor DNA template containing the target gene and a puromycin resistance cassette to promote DNA repair via homologous recombination. Following 2 weeks of puromycin selection, positive clones were selected from the cell pools through polymerase chain reaction (PCR) for the Puro-GFP insert and validated by Sanger sequencing.

**Figure 1:**
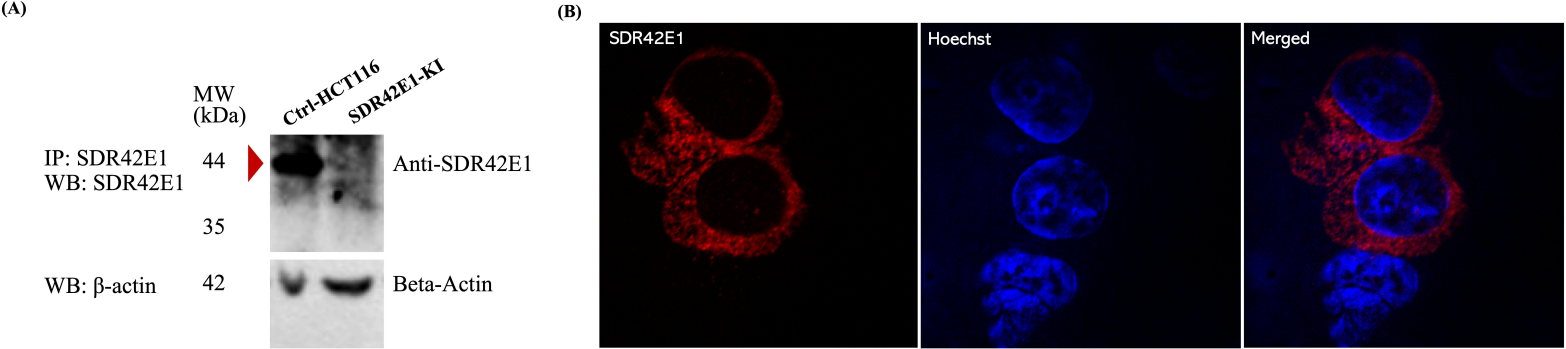
Localization and expression of SDR42E1 protein in HCT116 cells. **(A)** HCT116 cells, expressing wild-type SDR42E1 (green), were transiently transfected with 10 µg of SDR42E1-HA plasmid and stained overnight with a rabbit SDR42E1-tag polyclonal antibody (1:100 dilution). Nuclear staining was done by Hoechst staining (blue). **(B)** Western blot (WB) of immunoprecipitated (IP) SDR42E1 from wild-type control HCT116 cells (Ctrl-HCT116) detected a 44 kDa SDR42E1 protein, indicated with a red arrow, which was absent in the *SDR42E1*-knockin model (SDR42E1-Ki) using a rabbit polyclonal antibody against SDR42E1 (PA5-53156, Invitrogen). Before conducting the IP, a fraction (one-tenth) of the total lysates were analyzed via WB as input controls with a monoclonal anti-beta-actin mouse antibody (A5441, Sigma, 1:5000 dilution). All experimental conditions were analyzed with three independent biological replicates (n = 3 per group).

The overall CRISPR editing process, including gRNA design, donor synthesis, and screening of up to 100 single-cell clones, was carried out by GenScript. Editing efficiency in the initial cell pool was confirmed by Sanger sequencing, with successful targeting further validated by Western blot and immunofluorescence to confirm loss of *SDR42E1* protein expression (**Figure 1**). GenScript’s quality control criteria included MycoAlert™ mycoplasma testing, cell viability assessment, and full allelic validation by sequencing.

### 2.4 RNA extraction and RNA sequencing

Total RNA was extracted from the *SDR42E1*-gene edited cells through 1 mL of TRIzol reagent (Invitrogen), following the protocol provided by the manufacturer. The integrity and quantity of the extracted RNA were assessed using agarose gel electrophoresis and a NanoDrop 8000 spectrophotometer (Thermo Scientific). Following this, 2 μg of the total RNA was converted into complementary DNA (cDNA) utilizing the High-Capacity cDNA Reverse Transcriptase Kit (Applied Biosystems, United States), adhering to the manufacturer’s instructions.

To explore differentially expressed genes within relevant pathways, equal quantities of high-quality RNA pools (100–200 ng) were precipitated using 75% ethanol and sent to Macrogen Inc. (http://macrogen.com, Korea) for constructing RNA-sequencing libraries and performing next-generation sequencing. The TruSeq Stranded Total RNA Library Prep Kit (Illumina) was used to prepare the RNA sequencing libraries, according to the manufacturer’s guidelines. After PCR enrichment, the integrity and quantity of the libraries were assessed before sequencing on the Illumina NovaSeq 6000 platform, which produced 101 bp paired end reads. The raw sequence data underwent trimming to remove PCR duplicates, contaminants, and adapter sequences. Low-quality reads (below Q20) were filtered out utilizing CLC Genomics Workbench version 22.0.2, which was also used for assembling and aligning the reads to the latest human genome release (*Homo sapiens*, GRCh38). Genes showing average raw counts below 10 were discarded.

Differential gene expression analysis between knock-in and control samples identified significant changes characterized by at least a 2-fold increase or decrease in expression and an adjusted *P*-value of less than 0.05. This analysis was conducted using the R package DESeq2 version 1.34 with default settings (17). Heatmaps of gene expression data were generated using log_2_ fold-change maximum likelihood estimate (lfcMLE) values and −log_10_ of false discovery rate (FDR) values, and visualized using the R package pheatmap (version 1.0.2). The Pathview R package facilitated the visualization of KEGG pathways involving the differentially expressed genes (DEG) (18). GSE was carried out on human gene sets from the Enrichment map, ordered by the unshrunk log_2_ fold change values provided by DESeq2, as previously described (19). ClusterProfiler version 4.2.2, a tool known for its robust visualization capabilities, was used for functional annotation analysis utilizing standard parameters, including Gene Ontology (GO), Kyoto Encyclopedia of Genes and Genomes (KEGG) pathway, and enrichment analyses (20).

Validation of the RNA-sequencing results was performed through a comparative analysis between the RNA-sequencing data from the knock-in and overexpression of *SDR42E1* in HCT116 cells and genes linked to vitamin D traits in the NHGRI-EBI GWAS Catalog (EFO_0004631), with the catalog version released on July 08, 2024, and accessed on July 25, 2024 (21). This analysis focused on genetic markers within a 250-kilobase region of the GWAS signals and a statistical significance threshold of a *P*-value < 5.0 e−^8^. The aim was to thoroughly identify significant genes linked to vitamin D in our knock-in model, thereby enhancing the robustness of our research findings.

### 2.5 Cell lysis and Western blot of immunoprecipitation

To verify the loss of *SDR42E1* protein expression in gene-edited HCT116 cells, cells were lysed in ice-cold radioimmunoprecipitation assay (RIPA) buffer containing a protease inhibitor cocktail (UD282713, Thermo Fisher Scientific), dithiothreitol (1M), and phenylmethylsulfonyl fluoride (1 mM). The lysates were incubated for 30 minutes at 4°C with rotation, then centrifuged at 10,000 Xg for 15 minutes at 4°C. The clear supernatant was stored at −80°C. Protein concentrations were measured using a bicinchoninic acid assay kit (Pierce, Thermo Fisher Scientific).

To prepare for immunoprecipitation, cell extracts were mixed with an anti-SDR42E1 rabbit monoclonal antibody (WG3329739B, Invitrogen, 1:500 dilution) and rotated overnight at 4°C. Protein samples were then incubated with Protein A/G magnetic beads (Pierce, 88802, Thermo Scientific) for 2 hours at 4°C, followed by magnetic separation to remove unbound material. The beads were washed thrice with cold RIPA buffer and the proteins were eluted by heating in 5X SDS-sample buffer at 37°C for 30 minutes.

For Western blot analysis, an equal quantity of proteins was separated on a Novex NuPAGE 4-12% Bis-Tris SDS-polyacrylamide gel (Invitrogen) and wet-transferred onto a nitrocellulose membrane (Millipore). The membrane was blocked with 5% bovine serum albumin (BSA, Tocris, United Kingdom) for one hour at room temperature, then probed with the same anti-SDR42E1 antibody for three days at 4°C. Beta-actin was used as a loading control, detected with a monoclonal anti-beta-actin mouse antibody (1:5000 dilution, A5441, Sigma-Aldrich). After washing with Tris Buffered Saline with Tween 20 (TBST), the bands were visualized using a chemiluminescent substrate (Pierce ECL, Thermo Scientific) after application of peroxidase IgG fraction monoclonal anti-mouse IgG (H+L) secondary antibody HRP conjugate (1:5000 dilution, 62-6520, Invitrogen) or anti-rabbit IgG light chain specific (1:5000 dilution, 211-032-171, Jackson ImmunoResearch, United Kingdom).

### 2.6 Proteomics

For preparation, protein samples were incubated at 37°C for 30 minutes after the whole-cell extraction described earlier. Each sample, comprising 50 μg of protein from three biological replicates of the *SDR42E1* knock-in and HCT116 control samples, was then loaded onto a Novex NuPAGE 4-12% Bis-Tris SDS-polyacrylamide gel and electrophoresed at 100 volts for about 60 minutes. Overnight staining of the proteins was performed using PageBlue Protein Staining Solution at 4°C, and the gel was subsequently washed for 10 minutes with sterilized distilled water. Each gel lane was excised, placed in individual tubes, and stored at 4°C for subsequent mass spectrometry analysis as detailed in a previous study (22).

Protein samples were treated with dithioerythritol and s-carbamidomethylation with iodoacetamide as reducing agents prior to in-gel tryptic digestion. The gel pieces were washed and rehydrated with sequencing-grade modified porcine trypsin and incubated overnight at 37°C. The peptides were then extracted, desalted, and loaded onto a nanoflow ultra-performance liquid chromatography (UPLC) system, where they were separated using a gradient elution. Analysis of the resulting peptides was performed through an Orbitrap Fusion Tribrid mass spectrometer (Thermo Scientific). The relative abundance of proteins was determined by measuring the areas of precursor ions from unique, non-conflicting peptides. Data was then analyzed by R packages Limma (Linear Models for Microarray Data) version 3.56.2, Mascot Daemon (version 2.6.0, Matrix Science), and Progenesis QI (Version 2.2., Waters). Benjamini–Hochberg approach was utilized to convert Student’s t-tests-derived *P*-values to multiple test-corrected q-values, with a cut-off of <0.05. Functional annotations and enrichment Analysis were conducted on the ranked proteins based on *P*-values against *Homo sapiens* protein sets through ClusterProfiler.

### 2.7 Cell viability assay

To assess the cell viability of *SDR42E1* knock-in compared to wild-type HCT116, cells (2e^4^ cells/mL) were seeded in 96-well plates with a complete medium. Two-fold serial dilutions were performed, yielding a final concentration of around 6000 cells/mL, with control wells containing only a complete medium. Viability was assessed at 24–48 and 72 hours of incubation using the CellTiter-Glo^®^ Luminescent Cell Viability (Promega Corporation, G755A, USA) per manufacturer’s protocol. The assay, measuring ATP levels, provides a luminescent signal proportional to the percentage of living cells.

### 2.8 Immunofluorescence

Wild-type HCT116 cells were seeded on poly-L-lysine-coated coverslips within a 12-well plate. Once the cells reached 40-60% confluency, they were transiently transfected with 5 micrograms of SDR42E1-HA tag plasmids (Addgene) through Lipofectamine 3000 (2293283, Thermo Fisher Scientific), following the provided guidelines for 24 hours. Post-transfection, the cells were fixed with 4% paraformaldehyde and then blocked with 5% BSA in DPBS with 0.1% Tween-20 for 60 minutes at room temperature. After washing with DPBS, the cells were incubated with primary antibodies overnight at 4°C, followed by repeated washes in DPBS and 60-minute incubations with both secondary and tertiary antibodies sequentially at room temperature.

Primary antibodies, including recombinant rabbit HSP60 (Heat shock protein 60)-mitochondrial marker monoclonal antibody (HSPD1-2206R, Thermo Scientific), rabbit calreticulin-endoplasmic reticulum marker IgG polyclonal antibody (PA3900, Thermo Scientific), and mouse monoclonal anti-Golgi 58K antibody (NB600-41, Invitrogen), were used at a 1:100 dilution. Secondary antibodies, including biotinylated goat anti-rabbit IgG (H+L) (65-6140, Thermo Scientific) and biotinylated goat anti-mouse IgG (BA-9200, Vector), and tertiary antibody, Streptavidin Alexa Fluor 488 conjugate (S11223, Thermo Scientific), were applied at a 1:250 dilution. The nuclei were stained using Hoechst (33258, Invitrogen) at 1 µg/ml, and slides were mounted using ProlongTM Antifade Histomount (Thermo Fisher Scientific). Confocal microscopy images were conducted at 100X magnification using consistent imaging parameters.

### 2.9 Confocal microscopy

Microscope images were captured utilizing the Nikon A1R confocal fluorescence microscope with motor arm set at 60 μm, 0.3 mm, and 6.0 mm to ensure consistent field visualization. Image reconstruction and analysis were conducted through Fiji-Image J software (23). All images received uniform treatment to eliminate any potential bias.

### 2.10 Statistical analyses

Statistical analyses were conducted through GraphPad Prism 10. Two-group comparisons were evaluated using two-tailed unpaired or paired Student’s t-test, while multiple group comparisons utilized Kruskal-Wallis one-way ANOVA with Dunn’s *post hoc* test. Statistical significance was identified at a *P*-value threshold of 0.05. Findings are expressed as mean ± standard deviation or ± standard error of the mean (SEM), with a minimum of three independent biological replicates per experiment.

## 3 Results

### 3.1 Localization of SDR42E1 to the cytoplasmic membrane in HCT116 cells

To analyze the expression and cellular distribution of the wild-type SDR42E1, we transiently introduced a custom-constructed plasmid containing the full *SDR42E1* coding sequence into HCT116 cells. Western blot confirmed the SDR42E1 expression at the expected 44 kDa molecular weight (**Figure 1A**), and cellular localization assessments revealed a major presence in the cytoplasm and on the cell membrane (**Figure 1B**), with no detection in the endoplasmic reticulum, Golgi apparatus, and mitochondria (data not shown).

### 3.2 Transcriptomic profiling reveals vitamin D regulation mechanisms in SDR42E1 knock-in model

To investigate the functional role of *SDR42E1* in HCT116 cells, a homozygous *SDR42E1* knock-in model was generated using the CRISPR/Cas9 gene-editing technology, incorporating the rs11542462 non-sense mutation. Prior to the functional investigations, the complete absence of *SDR42E1* expression was confirmed through Western blotting on immunoprecipitated SDR42E1 in whole cell lysates (**Figure 1A**).

**Figure 2:**
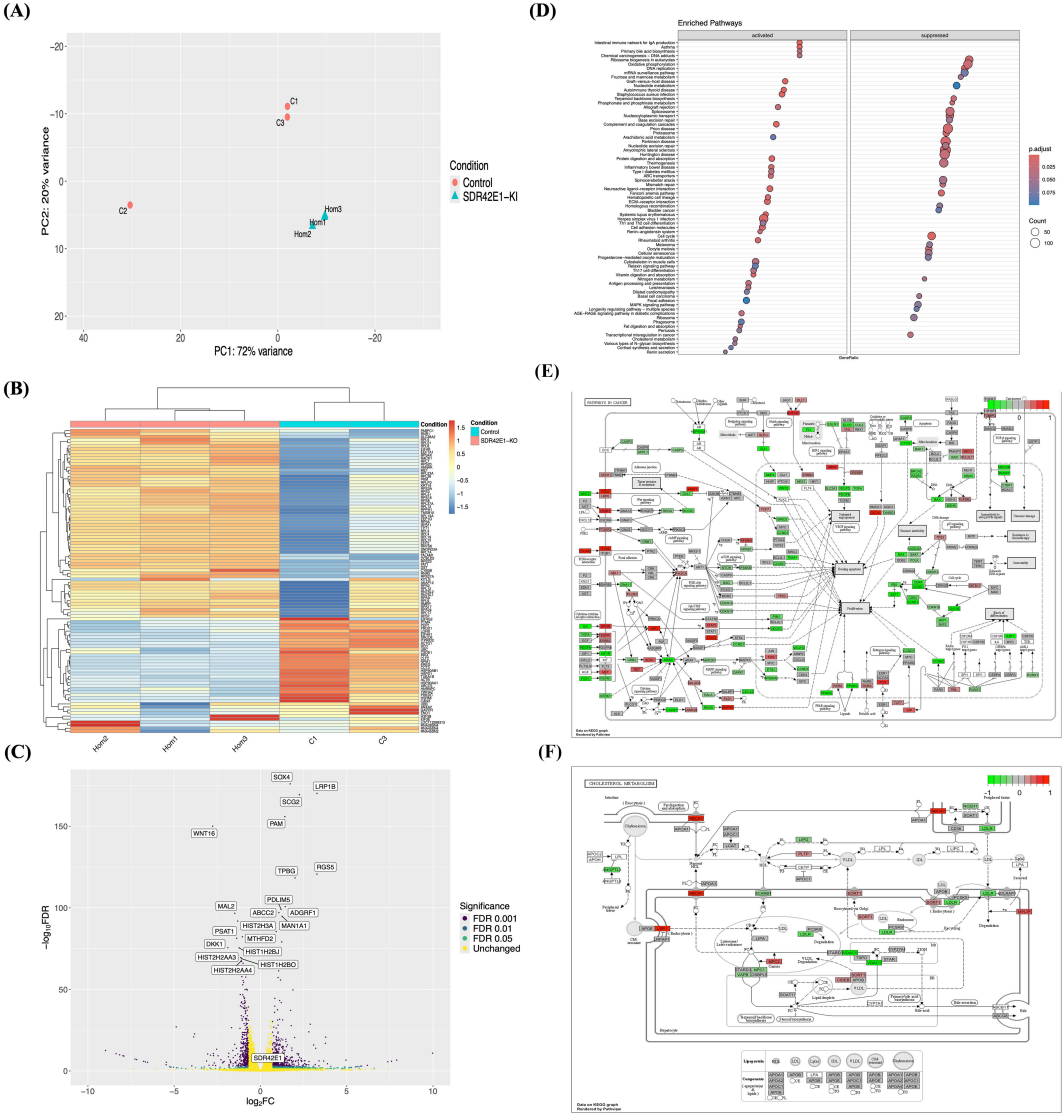
Transcriptomic reveals vitamin D regulation mechanisms in *SDR42E1* knock-in model. **(A)** PCA plot shows clustering of wild-type HCT116 controls (C; in rose) and *SDR42E1* knock-in (Hom, in blue) biological replicates, along the major principal components of the regularized log-transformed counts. **(B)** Heatmap of top 100 DEGs displays the Z-scores of regularized log-transformed counts with lower (blue) and higher (red) expressions. The X- and Y-axes are labeled with sample names and DEGs, respectively. **(C)** Volcano plot of significant gene expression changes in *SDR42E1* knock-in compared to wild-type controls. The X-axis represents the log_2_ fold change (FC), with upregulated genes to the right and downregulated genes to the left with Log_2_FC > 2 or < −2, while the Y-axis shows the false discovery rate (FDR) < 0.05, with the top 20 most significantly altered genes labeled. **(D)** Dot plot shows KEGG pathway enrichment of DEGs, with KEGG pathways on the Y-axis and gene ratio on the X-axis. KEGG pathways diagram shows genes expression profiles **(E)** in pathways of cancer, and **(F)** in cholesterol metabolism with upregulated (red) and downregulated (green) genes. All analyses were performed and visualized using R/DESeq2, Pheatmap, or Pathview. All experimental conditions were analyzed with three independent biological replicates (n = 3 per group).

RNA-sequencing analysis using DESeq2/R revealed 4,663 DEG among 19,285 total reads, with an adjusted *P*-value FDR below 0.05 compared to wild-type controls (**Figure 2**). Principal component analysis (PCA) and multidimensional scaling (MDS) successfully distinguished the *SDR42E1* knock-in samples from the wild-type cells (**Figure 2A**, **Supplementary Figure S2a**), a distinction further supported by a heatmap showcasing the top 100 DEG (**Figure 2B**).

A volcano plot demonstrated approximately 56% of DEGs were upregulated with a log2 fold change (FC) ≥ 0.3 (**Figure 2C**), including SRY-box transcription factor 4 (*SOX4*, FC = 1.7, FDR < 1.0 e−170), low-density lipoprotein receptor-related protein 1B (*LRP1B*, FC = 3.3, FDR < 1.0e−160), regulator of G protein signaling 5 (*RGS5*, FC = 3.3, FDR < 1.0e−100), and ATP-binding cassette C2 (*ABCC2*, FC = 1.10, FDR < 1.0e−90). Further, 525 DEG were efficiently downregulated with a log2 FC of ≤ −0.3, including wingless-type MMTV integration site 16 (*WNT16*, FC = −2.8, FDR < 1.0e−140), histone cluster 2H3A (*HIST2H3A*, FC = −1.31, FDR < 1.0e−80), *SLC7A5* (FC = −1.07, FDR < 1.0e−50), and aldolase A (*ALDOA*, FC = −0.37, FDR < 1.0e−04) (**Supplementary Table S2**).

Functional enrichment analysis of DEG revealed the biological processes influenced by *SDR42E1* knock-in in HCT116 cells. KEGG pathway analysis through the ClusterProfiler/R identified several activated pathways, including immunity disorders (FC = 2.6, adjusted *P*-value < 1.0e−04), vitamin digestion and absorption (FC = 2.0, adjusted *P*-value < 1.0e−03), ABC transporters (FC = 1.8, adjusted *P*-value < 1.00e−02), inflammatory bowel disease (FC = 1.8, adjusted *P*-value < 1.0e−02), and primary bile acid biosynthesis (FC = 1.6, adjusted *P*-value < 1.0e−02), while pathways related to DNA repair and replication (FC = −2.4, adjusted *P*-value < 1.0e−04), cell cycle regulation (FC = −2.5, adjusted *P*-value < 1.0e−04), transcriptional misregulation in cancer (FC = −1.7, adjusted *P*-value < 1.0e−02), and cellular senescence (FC = −1.5, adjusted *P*-value < 1.0e−02) were deactivated (**Figure 2D**).

Specific gene alterations in cancer and cellular senescence were noted, including downregulation of wingless-type MMTV integration site 2 (*WNT2*, FC = −2.8, FDR < 1.0e−140) and heat shock protein 90kDa alpha (cytosolic) A1 (*HSP90AA1*, FC = −0.67, FDR < 2.0e−20), and upregulation in signal transducer and activator of transcription 2 (*STAT2*, FC = 0.6, FDR < 5.0e−08) and human leukocyte antigens A (*HLA-A*, FC = 0.38, FDR < 3.0e−05) (**Figure 2F**, **Supplementary Figure S2b**). Additionally, low-density lipoprotein receptor (*LDLR*, FC = −0.56, FDR < 2.0e−11), cytochrome P450 enzymes such as *CYP51A1* (FC = −0.44, FDR = 3.0e−09), and 24-dehydrocholesterol reductase (*DHCR24*, FC = −0.6, FDR < 1.0e−14) were downregulated, while lipoprotein receptor related protein (*LRP1*, FC = 3.3, FDR < 5.0e−160) and *ABCA1* (FC = 1.5, FDR < 1.0e−06) were upregulated in cholesterol metabolism and steroid biosynthesis (**Figure 2E**, **Supplementary Figure S2c**).

Moreover, gene set enrichment function analysis (GSE) confirmed pathways linked to protein-lipid complex remodeling (FC = 1.9, adjusted *P*-value < 2.0e−03), sterol transfer and transporter activities (FC = 1.8, adjusted *P*-value < 5.0e−03), intestinal lipid and sterol absorption (FC = 1.9, adjusted *P*-value < 5.0e−03), lipid digestion (FC = 1.7, adjusted *P*-value < 1.0e−02), ATPase-coupled lipid transmembrane transporter activity (FC = 1.6, adjusted *P*-value < 1.0e−02), and cholesterol metabolic process (FC = 1.5, adjusted *P*-value < 4.0e−02). Downregulated pathways included sterol biosynthesis (FC = −1.9, adjusted *P*-value < 1.1e−02), osteoblast development (FC = −1.6, adjusted *P*-value < 1.0e−02), and NADH dehydrogenase activity (FC = −1.7, adjusted *P*-value < 4.0e−02) (**Supplementary Figure S2d**).

To validate our findings, we conducted a comparative analysis of DEG from the *SDR42E1* knock-in model with vitamin D-related GWAS Catalog data (21). This analysis revealed that 50 out of 304 genes were replicated in both datasets. Among these, key genes such as transcription factor Dp-2 (*TFDP2*), carbamoyl-phosphate synthase 1 (*CPS1*), farnesyl diphosphate synthase (*FDPS*), *CYP24A1*, and *LDLR* showed significant correlations, with *P*-values ranging from 2e−342 to 5e−08 (**Table 1**).

**Table 1:** Replication of differentially expressed genes reported in GWAS catalog for vitamin D in the SDR42E1 knock-in model.

Gene	Base Mean	log_2_ FC	*P*-value	FDR	Gene Description
*TFDP2*	1900.00	0.98	3.24e−38	7.56e−36	Transcription Factor Dp-2, regulates cell cycle genes by complexing with E2F proteins.
*CPS1*	1774.78	0.64	9.23e−19	6.65e−17	Carbamoyl-Phosphate Synthetase 1, catalyzes carbamoyl phosphate formation in the urea cycle.
*FDPS*	1833.00	−0.60	8.43e−18	5.43e−16	Farnesyl Diphosphate Synthase, synthesizes farnesyl diphosphate for sterol and isoprenoid biosynthesis.
*CYP24A1*	1738.07	−0.63	3.82e−14	1.49e−12	Cytochrome P450 24A1, catabolizes vitamin D and regulates calcium homeostasis.
*LTA4H*	4696.20	0.48	2.57e−13	8.91e−12	Leukotriene A4 Hydrolase, converts leukotriene A4 to B4, involved in inflammation.
*LDLR*	1423.96	−0.55	6.32e−13	2.03e−11	Low-Density Lipoprotein Receptor, mediates LDL endocytosis, crucial for cholesterol balance.
*USP1*	1270.99	−0.70	1.88e−12	5.63e−11	Ubiquitin-Specific Protease 1, regulates DNA repair and cell cycle through deubiquitination.
*KIF20B*	1872.81	−0.50	4.93e−11	1.22e−09	Kinesin Family Member 20B, motor protein essential for mitotic spindle and cytokinesis.
*NRIP1*	2328.67	0.43	3.97e−10	8.52e−09	Nuclear Receptor Interacting Protein 1, coactivator modulating nuclear receptor activity.
*PSMA1*	3088.24	−0.37	5.00e−09	8.86e−08	Proteasome Subunit Alpha 1, component of the 20S proteasome, involved in protein degradation.
*ABCA1*	120.90	1.35	8.70e−08	1.24e−06	ATP-Binding Cassette Subfamily A Member 1, transports cholesterol and phospholipids for HDL formation.
*PIP5K1A*	1272.09	−0.38	1.33e−06	1.46e−05	Phosphatidylinositol-4-Phosphate 5-Kinase 1 Alpha, synthesizes PIP2 for signaling and actin dynamics.
*MMADHC*	2342.79	−0.31	1.67e−06	1.79e−05	Methylmalonic Aciduria and Homocystinuria, cobalamin-Related Disorder, Involved in vitamin B12 biosynthesis.
*DHCR7*	864.44	−0.44	2.13e−06	2.24e−05	7-Dehydrocholesterol Reductase, converts 7-dehydrocholesterol to cholesterol.
*RETREG3*	1082.38	0.36	9.98e−06	9.05e−05	Reticulon 3, involved in ER morphology and stress responses.
*PFDN4*	557.22	−0.49	1.03e−05	9.29e−05	Prefoldin 4, chaperone assisting in protein folding and aggregation prevention.
*GPAM*	1087.02	−0.36	1.37e−05	0.0001	Glycerol-3-Phosphate Acyltransferase Mitochondrial, synthesizes glycerolipids, crucial for energy metabolism.
*RHOA*	6678.37	−0.22	1.49e−05	0.0001	Ras Homolog Family Member A, regulates actin cytoskeleton and cell motility.
*SDR42E1*	834.62	0.41	2.88e−05	0.0002	Short-Chain Dehydrogenase/Reductase Family 42E Member 1, involved in steroid metabolism.
*KLHL8*	1005.10	−0.37	2.92e−05	0.0002	Kelch-Like Protein 8, Adapter in ubiquitin ligase complexes, affecting protein degradation.
*HSD17B11*	1079.96	0.28	0.0005	0.0027	Hydroxysteroid Dehydrogenase 17 Beta 11, metabolizes steroid hormones and regulates endocrine functions.
*NADSYN1*	415.27	0.35	0.0022	0.0103	Nicotinate Adenine Dinucleotide Synthetase 1, biosynthesizes NAD, crucial for metabolism.

Genes significantly associated with vitamin D levels as identified from RNA-sequencing of the *SDR42E1* knockin HCT116 model and reported in the GWAS Catalog (*P*-values < 5e−08). FDR, *P*-value adjusted using Benjamini-Hochberg in DESeq2.

### 3.3 Proteomic insights validate extensive pathway modifications upon SDR42E1 depletion

**Figure 3:**
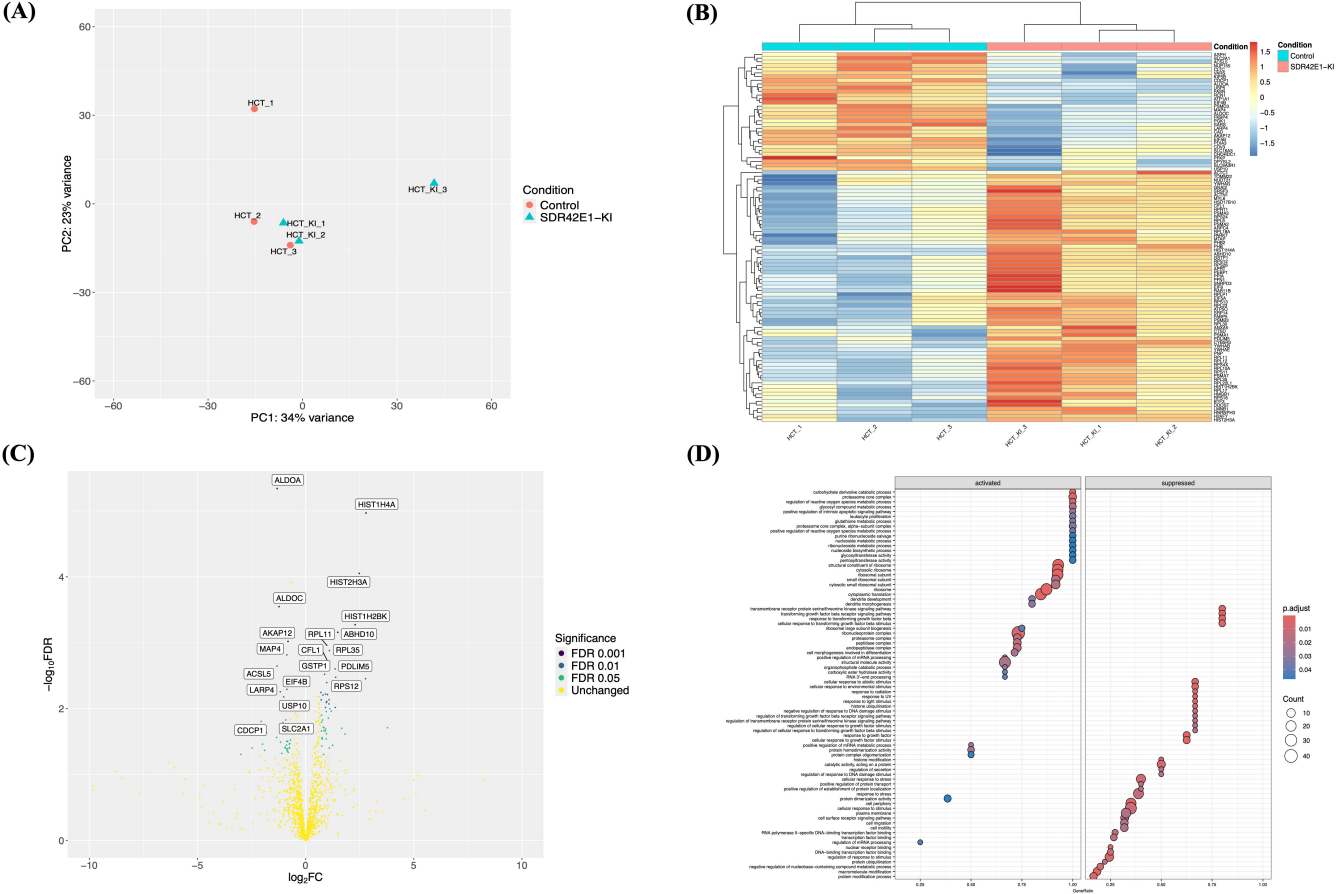
Proteomic insights validate extensive pathway modifications upon *SDR42E1* depletion. **(A)** PCA plot of wild-type HCT116 controls (HCT; in rose) and *SDR42E1* knock-in (HCT_KI, in blue) based on the major components of regularized log-transformed counts. **(B)** Heatmap of top 100 differentially expressed proteins displays the Z-scores of log-transformed counts, with lower (blue) and higher (red) expression. Axes are labeled with sample names and proteins. **(C)** Volcano plot of proteomic data comparing *SDR42E1* knock-in to wild-type HCT116 cells. The X-axis shows log_2_ fold change (FC), with significant upregulation to the right (greater than 2) and downregulation to the left (less than −2), while the Y-axis shows the −log of the false discovery rate (FDR), marking significance below 0.05. The top 20 significantly altered proteins are labeled. **(D)** GSE dot plot of pathway enrichment analysis of the differentially expressed proteins in *SDR42E1* knock-in. These analyses were conducted and visualized using R/Limma, Pheatmap, and ClusterProfiler. All experimental conditions were analyzed with three independent biological replicates (n = 3 per group).

To validate the transcriptomic profile and identify differentially expressed proteins in HCT116 cells harboring the homozygous *SDR42E1* knock-in non-functional variant, three replicates of *SDR42E1* knock-in and wild-type cells underwent label-free LC-MS/MS analysis. Differential protein expression was assessed using a Student’s t-test in the Limma R package, with a significance threshold of *P*-value < 0.05. Proteomic analysis revealed substantial differences in protein profiles between *SDR42E1* knock-in and wild-type HCT116 cells, as demonstrated by the PCA analysis (**Figure 3A**). This finding was further supported by heatmap and volcano plots (**Figures 3B, C**), highlighting 140 differentially expressed proteins out of 1,320 analyzed in the knock-in model (**Supplementary Table S3**).

Several proteins showed consistent regulation with RNA-sequencing findings. Specifically, downregulated proteins included aldolase fructose-bisphosphate A (ALDOA, *P*-value = 4.56e−06, Log2FC = −1.32), fatty acid synthase (FASN, *P*-value = 4.56e−04, Log2FC = −1.32), aldolase fructose-bisphosphate C (ALDOC, *P*-value = 2.80e−04, Log2FC = −1.23), A kinase (PRKA) anchor protein 12 (AKAP12, *P*-value = 9.50e−04, Log2FC = −0.90), and acyl-CoA synthetase long chain family 5 (ACSL5, *P*-value = 2.20e−03, Log2FC = −1.32). Upregulated proteins, included histone 1 H4A (HIST1H4A, *P*-value = 1.07e−05, Log2FC = 2.80), HIST2H3A (*P*-value = 8.90e−05, Log2FC = 2.50), abhydrolase domain containing 10 (ABHD10, *P*-value = 6.90e−04, Log2FC = 2.80), ribosomal protein L11 (RPL11, *P*-value = 1.00e−03, Log2FC = 2.80), and H2A Histone Family, Member Y (H2AFY, *P*-value = 3.50e−03, Log2FC = 2.76) (**Figure 3C**).

Pathway enrichment analysis highlighted several key processes, including the upregulation of ribosomal structure (FC = 2.14, adjusted *P*-value = 2.70e−04), carbohydrate derivative catabolic process (FC = 1.8, adjusted *P*-value = 6.00e−04), cytoplasmic translation (FC = 1.90, adjusted *P*-value = 1.30e−03), metabolic process (FC = 1.71, adjusted *P*-value = 8.40e−03), and reactive oxygen species process (FC = 1.69, adjusted *P*-value = 8.90e−03). Downregulated pathways involved response to growth factor (FC = −2.06, adjusted *P*-value = 7.50e−04), cell periphery (FC = 2.14, adjusted *P*-value = 2.60e−03), transmembrane receptor protein serine/threonine kinase signaling pathway (FC = −1.90, adjusted *P*-value = 2.80e−03), and DNA repair (FC = −2.28, adjusted *P*-value = 9.10e−03) (**Figure 3D**).

### 3.4 Transcriptomic profiling unveils divergent pathway modifications upon SDR42E1 overexpression

**Figure 4:**
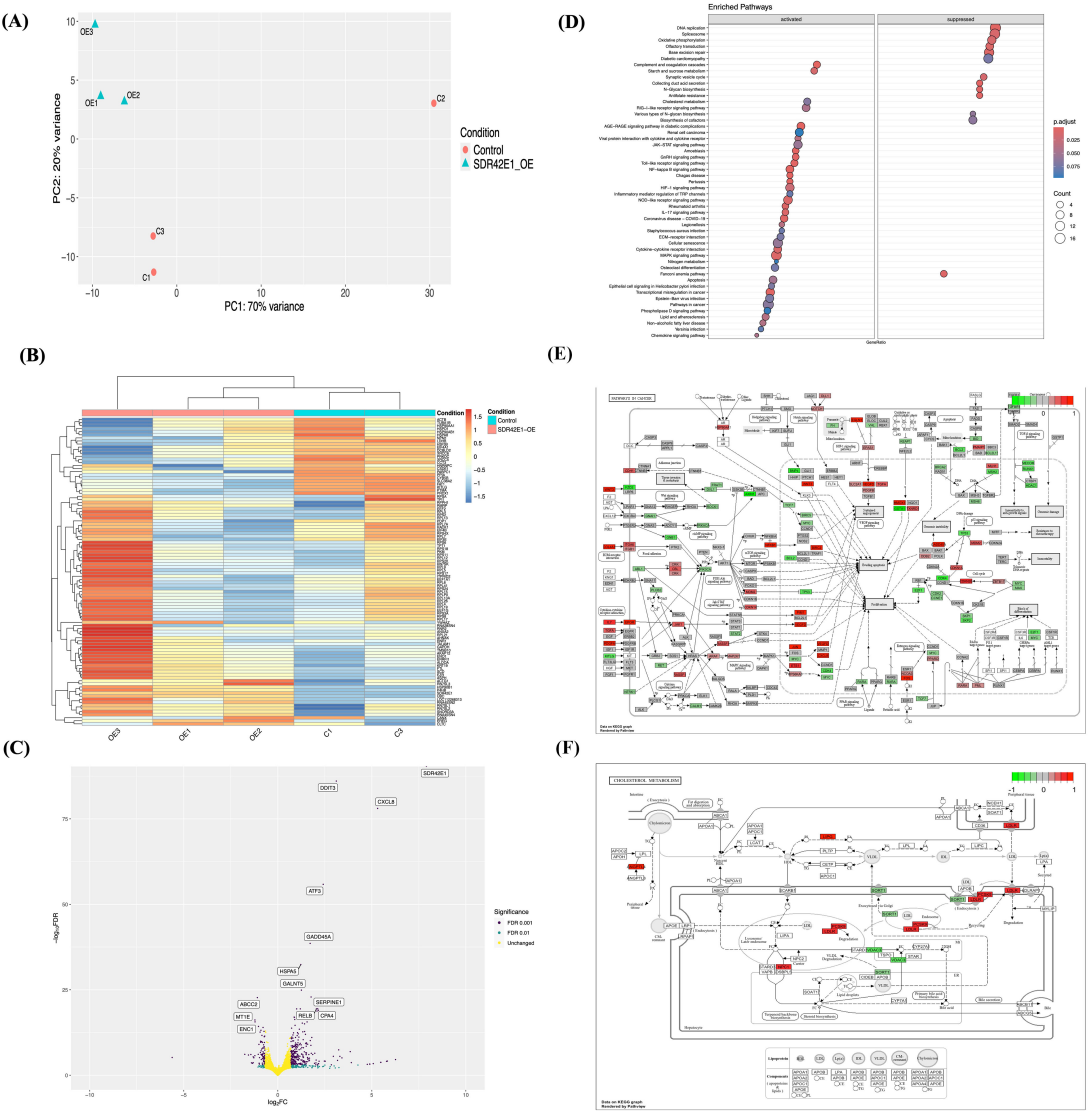
Transcriptomic unveils divergent pathway modifications upon *SDR42E1* overexpression. **(A)** PCA plot of wild-type HCT116 controls (C; in rose) and *SDR42E1* overexpression (Hom; in blue) replicates, with main principal components derived from regularized log-transformed counts. **(B)** Heatmap of top 100 DEGs shows Z-scores of regularized log-transformed counts, with blue indicating lower expression and red indicating higher expression distinguishing sample conditions. The X- and Y-axes are annotated with sample names and DEGs, respectively. **(C)** Volcano plot of significant gene expression changes in *SDR42E1* overexpression compared to wild-type controls. The X-axis represents log_2_ fold change (FC), with upregulated genes on the right and downregulated genes on the left (Log_2_FC > 2 or < −2), while the Y-axis indicates the false discovery rate (FDR) < 0.05, with the top 20 most significantly altered genes labeled. **(D)** Dot plot of KEGG pathway enrichment for DEGs, with the Y-axis showing KEGG pathways and the X-axis indicating genes ratio. KEGG pathway shows gene expression profiles involved **(E)** in cancer pathways, and **(F)** in cholesterol metabolism, with upregulated (red) and downregulated (green) genes. All analyses were performed and visualized using R/DESeq2, Pheatmap, and Pathview. All experimental conditions were analyzed with three independent biological replicates (n = 3 per group).

To understand the role of *SDR42E1* in cellular metabolism, we established a comparative baseline model through transiently overexpressing the *SDR42E1* gene in HCT116 cells and examined its impact on biological profiles and sterol pathways. The overexpression was achieved using a wild-type plasmid tagged with HA (**Figure 1A**) and was further confirmed via RNA-sequencing analysis. This analysis revealed significant transcriptional deregulations, with 1,483 DEG out of 19,285 total reads at an FDR < 0.05 compared to the control group, as demonstrated by PCA and MDS analysis (**Figure 4A**, **Supplementary Figure S3a**). This distinction was further corroborated by a heatmap showing the top 100 DEG (**Figure 4B**).

The majority of these genes, approximately 343 in total, showed increased expression levels, including *SDR42E1* (FC = 8.0, FDR < 2.2e−308), DNA damage-inducible transcript 3 (*DDIT3*, FC = 3.1, FDR = 6.12e−83), C-X-C motif chemokine ligand 8 (*CXCL8*, FC = 5.3, FDR = 4.1e−75), growth arrest and DNA damage-inducible Alpha (*GADD45A*, FC = 1.7, FDR = 2.7e−39), heat shock protein A (Hsp70) 5 (*HSPA5*, FC = 1.2, FDR = 9.85e−30), and serine protease inhibitor E1 (*SERPINE1*, FC = 1.7, FDR = 2.30e−20). Additionally, 95 genes exhibited decreased expression, including *ABCC2* (FC = −1.10, FDR = 3.05e−20), metallothionein 1E (*MT1E*, FC = −1.22, FDR = 4.35e−14), ectodermal-Neural Cortex 1 (*ENC1*, FC = −1.21, FDR = 1.49e−13), inhibitor of DNA binding 3 (*ID3*, FC = −1.03, FDR = 2.70e−12), and SRY-box transcription factor 4 (*SOX4*, FC = −0.78, FDR = 5.23e−12) (**Figure 4C**, **Supplementary Table S4**).

To explore the biological functions influenced by DEG in the *SDR42E1* overexpression model, we conducted a KEGG enrichment analysis that revealed activation in pathways related to nuclear factor kappa B (NF-kB) signaling pathway (FC = 2.2, adjusted *P*-value = 1.3e−04), transcriptional misregulation in cancer pathways (FC = 2.02, adjusted *P*-value = 1.8e−03), immune response pathway (FC = 1.9, adjusted *P*-value = 2.0e−03), cellular senescence (FC = 1.7, adjusted *P*-value = 2.0e−02), atherosclerosis and lipid (FC = 1.7, adjusted *P*-value = 3.0e−02), and cholesterol metabolism (FC = 1.6, adjusted *P*-value = 3.0e−02). In contrast, there was downregulation in the DNA repair and replication (FC = −2.2, adjusted *P*-value = 2.0e−03), collecting duct acid secretion (FC = −1.9, adjusted *P*-value = 1.7e−03), N-glycan biosynthesis (FC = −1.9, adjusted *P*-value = 3.0e−04), and antifolate resistance (FC = −1.9, adjusted *P*-value = 5.0e−03) (**Figure 4D**).

Notable genetic alterations were found within the KEGG pathways related to cancer, cellular senescence, cholesterol metabolism, and steroid biosynthesis. Critical genes in the cancer and cellular senescence, encompassing elevated expression of *CXCL8*, *GADD45*, jun proto-oncogene (*JUN*, FC = 1.3, adjusted *P*-value = 3.2e−13), and laminin beta 3 (*LAMB3*, FC = 1.4, adjusted *P*-value = 6.6e−11), as well as decreased tyrosine-kinase receptor G (*KITG*, FC = −0.57, adjusted *P*-value = 3.1e−06), retinoid X receptor alpha (*RXRA*, FC = −0.57, adjusted *P*-value = 1.4e−05), and axis inhibition protein 2 (*AXIN2*, FC = −0.93, adjusted *P*-value = 2.0e−05) (**Figure 4E**, **Supplementary Figure S3b**). In cholesterol metabolism and steroid biosynthesis, low-density lipoprotein receptor (*LDLR*, FC = 0.7, FDR = 4.1e−07), 7-dehydrocholesterol reductase (*DHCR7*, FC = 0.5, adjusted P-value = 1.1e−03), proprotein convertase subtilisin/kexin type 9 (*PCSK9*, FC = 0.6, FDR = 1.0e−03), and lipase G (*LIPG*, FC = 0.89, adjusted *P*-value = 1.7e−03) show upregulation, while *CYP24A1* (FC = −0.9, adjusted *P*-value = 7.0e−14), voltage-dependent anion channel (*VDAC3*, FC = −0.4, adjusted *P*-value = 4.3e−03), and sortilin (*SORT1*, FC = −0.4, adjusted *P*-value = 1.7e−03) exhibit downregulation (**Figure 4F**, **Supplementary Figure S3c**).

GSE function analysis also highlighted several enriched categories, including the activation of the inflammatory response (FC = 2.1, adjusted *P*-value = 1.1e−04), negative regulation of catalytic activity and molecular function (FC = 2.0, adjusted *P*-value = 2.0e−04), defense response (FC = 3.1, adjusted *P*-value = 2.0e−04), regulation of cell population proliferation (FC = 1.7, adjusted *P*-value = 5.0e−04), regulation of angiogenesis and vasculature development (FC = 2.1, adjusted *P*-value = 5.9e−04), steroid biosynthetic and metabolic processes (FC = 2.1, adjusted *P*-value = 1.0e−03), steroid dehydrogenase activity (FC = 1.7, adjusted *P*-value = 2.3e−03), and intracellular sterol transport (FC = 1.8, adjusted *P*-value = 4.0e−03). Deactivated categories included a response to lectin (FC = −2.24, adjusted *P*-value = 2.5e−04), condensed chromosome-centromeric region (FC = −2.5, adjusted *P*-value = 5.1e−04), mitochondrial respiratory chain complex assembly (FC = −2.29, adjusted P-value = 6.3e−04), folic acid binding and metabolic process (FC = −1.9, adjusted *P*-value = 1.4e−03), and NADH dehydrogenase activity (FC = −1.7, adjusted *P*-value = 4.0e−02) (**Supplementary Figure S3d**).

The replication of common genes was examined by comparing the DEG from the *SDR42E1* overexpressed HCT116 RNA-sequencing data with those associated with vitamin D in the GWAS Catalog. Our analysis identified a significant overlap, with 29 out of 304 genes showing replication in the *SDR42E1* overexpressed dataset. Prominent genes such as *SDR42E1*, C-X-C motif chemokine ligand 8 (*CXCL8*), *CYP24A1*, *LDLR*, *PCSK9*, and *LIPG* were prominently featured, showing strong associations in both datasets, with *P*-values spanning from 2e−342 to 5e−08 (**Table 2**).

**Table 2:** Replication of differentially expressed genes reported in the GWAS catalog for vitamin D in the *SDR42E1* overexpression model.

Gene	Base Mean	log_2_ FC	*P*-value	FDR	Gene Description
*SDR42E1*	105440.61	7.90	< 2.2e−308	<2.2e−308	Short Chain Dehydrogenase/Reductase Family 42E Member 1, metabolizes steroid hormones.
*CXCL8*	369.78	5.31	8.76e−79	4.10e−75	C-X-C Motif Chemokine Ligand 8, Cytokine that acts as a chemoattractant, involved in inflammation.
*HERPUD1*	988.64	0.92	2.56e−15	1.33e−12	Homocysteine-Responsive Endoplasmic Reticulum-Resident Ubiquitin-Like Domain 1, handles ER protein degradation.
*CYP24A1*	1682.61	−0.90	7.01e−14	3.08e−11	Cytochrome P450 Family 24 Subfamily A Member 1, degrades active vitamin D.
*VGF*	187.25	1.47	1.08e−12	3.63e−10	VGF Nerve Growth Factor Inducible, neuropeptide involved in energy homeostasis and synaptic plasticity.
*LDLR*	2647.74	0.72	2.67e−09	4.17e−07	Low-Density Lipoprotein Receptor, regulates cholesterol uptake into cells.
*NRIP1*	1637.50	−0.51	3.83e−06	0.0002	Nuclear Receptor Interacting Protein 1, co-regulates nuclear receptors, affecting gene expression.
*MGAM*	44.91	1.58	6.42e−06	0.0003	Maltase-Glucoamylase (Alpha-Glucosidase), enzyme that breaks down maltose into glucose.
*PCSK9*	1035.97	0.55	2.89e−05	0.0011	Proprotein Convertase Subtilisin/Kexin Type 9, regulates cholesterol by degrading LDL receptors.
*LIPG*	335.40	0.89	5.32e−05	0.0017	Lipase G, Endothelial Type, hydrolyzes triglycerides in lipoproteins, aiding lipid metabolism.
*UNC5CL*	45.08	1.38	5.51e−05	0.0018	UNC5C Like Netrin Receptor, involved in cell signaling and axon guidance.
*FDPS*	2949.32	0.38	0.0002	0.0053	Farnesyl Diphosphate Synthase, key enzyme in cholesterol and isoprenoid biosynthesis.
*PEX10*	203.27	−0.63	0.0007	0.0120	Peroxisomal Biogenesis Factor 10, essential for peroxisome formation and lipid metabolism.
*DHCR7*	1412.43	0.47	0.0012	0.0177	7-Dehydrocholesterol Reductase, key in cholesterol biosynthesis.
*WIPI1*	383.19	0.49	0.0019	0.0250	WD Repeat Domain, Phosphoinositide Interacting 1, involved in autophagy and lipid metabolism.
*PIP5K1A*	1837.28	0.30	0.0022	0.0279	Phosphatidylinositol-4-Phosphate 5-Kinase Type 1 Alpha, regulates PIP5-bisphosphate production.
*HSD17B11*	864.98	−0.32	0.0029	0.0339	Hydroxysteroid 17-Beta Dehydrogenase 11, converts androgens and estrogens in steroid metabolism.

Genes significantly associated with vitamin D levels as identified from RNA-sequencing of the *SDR42E1* overexpression HCT116 model and reported in the GWAS Catalog (*P*-values < 5e−08). FDR, *P*-value adjusted using Benjamini-Hochberg in DESeq2.

### 3.5 Pronounced reduction in cell viability upon SDR42E1 depletion

**Figure 5:**
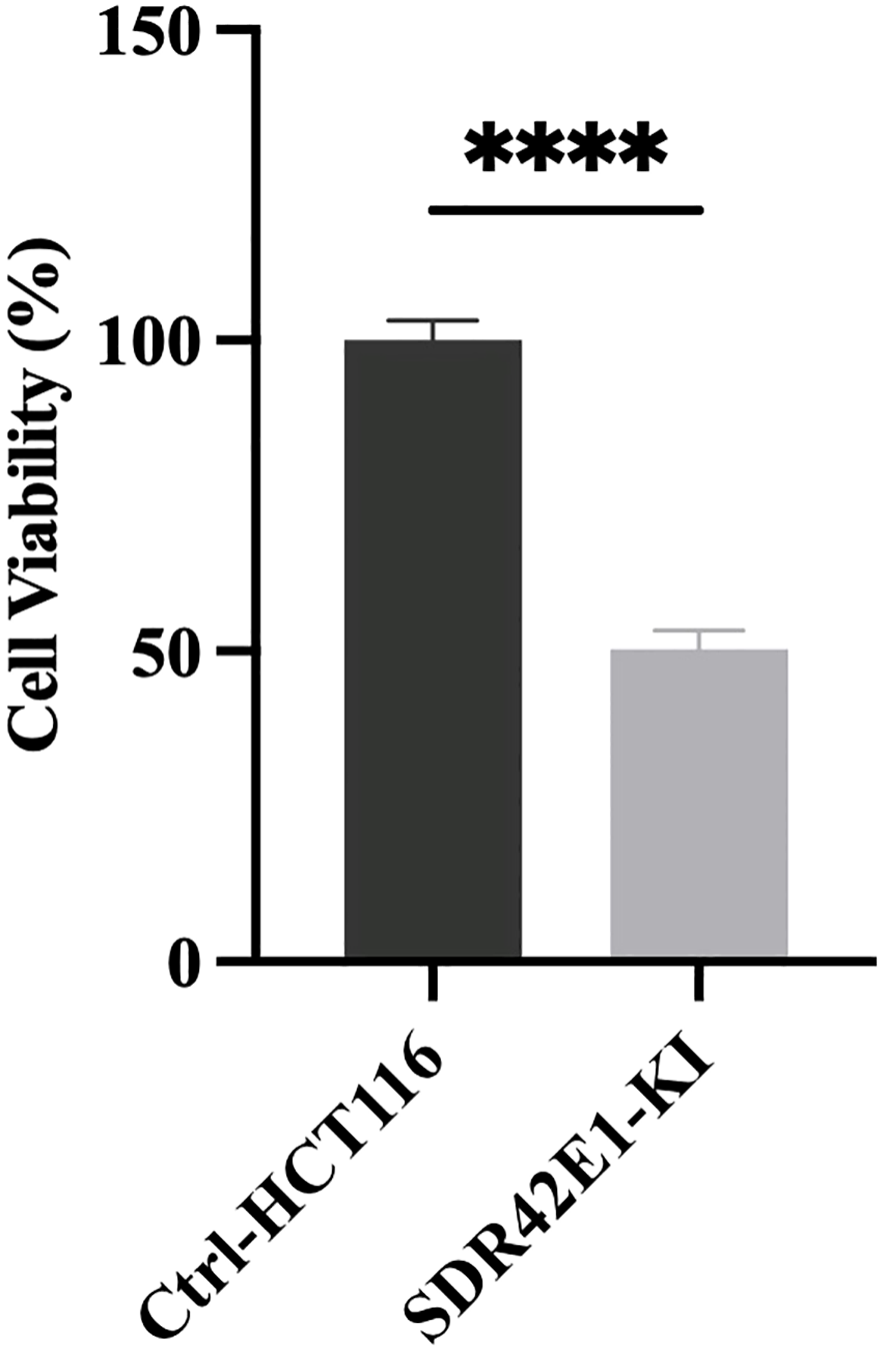
Pronounced reduction in cell viability upon *SDR42E1* depletion. Cell viability of the *SDR42E1* knock-in model was assessed using a CellTiter-Glo Luminescent Cell Viability (ATP) assay. Gene knock-in reduced viability compared to wild-type HCT116 cells among three replicate experiments (****P-value < 0.0001). All experimental conditions were analyzed with three independent biological replicates (n = 3 per group).

To explore the role of the SDR42E1 gene in cell viability, the survival of stable gene-edited HCT116 cells was assessed compared to wild-type controls using the CellTiter-Glo luminescent cell viability assay. The results indicated a significant decrease in cell viability of the *SDR42E1* knock-in HCT116 cells by 53%, with a *P*-value less than 1.0e−04 (**Figure 5**).

## 4 Discussion

Vitamin D deficiency is a widespread health concern, contributing to health disorders such as osteoporosis, rickets, cardiovascular diseases, and immune dysfunction (1). Understanding the complex mechanisms governing vitamin D absorption and metabolism is crucial for effective interventions. The absorption of lipids and steroids involves intricate processes, including bile acid micelles and intestinal transport proteins. Any defects or inefficiencies in these processes exacerbate the deficiency (24), emphasizing the importance of studying related genes and pathways.

Our study highlights *SDR42E1* as a key player in vitamin D absorption, a gene recently linked to serum 25(OH)D levels through GWAS research (12–14). *SDR42E1* is implicated in multiple metabolic processes that potentially affect lipid metabolism and steroid synthesis (9, 10). While SDR family proteins localize to diverse cellular compartments (9, 25), recent research has identified SDR42E1 in the plasma membrane and cytoplasm of keratinocytes, a crucial site for vitamin D biosynthesis (16). We confirmed significant cytoplasmic membrane localization of *SDR42E1* in intestinal HCT116 cells, supporting its role in lipid and steroid metabolism. Although our focus has been on genomic pathways, vitamin D also exerts rapid non-genomic effects through membrane-associated VDRs and calcium signaling cascades (26). Given SDR42E1’s predominant membrane localization, it is plausible that it modulates these non-canonical pathways—such as membrane-initiated rapid responses and calcium-mediated signaling—highlighting a broader functional impact that warrants further investigation.

CRISPR/Cas9-mediated *SDR42E1* nonsense mutation and the transient overexpression induced considerable transcriptomic changes in the HCT116 models. *LRP1B* and *ABCC2* were upregulated upon *SDR42E1* depletion but downregulated in overexpression models, highlighting their role in vitamin D and sterols absorption. *LRP1B*, a low-density lipoprotein receptor family member, facilitates the endocytosis of lipid-based vitamins (27). Similarly, *ABCC2*, an ATP-binding cassette transporter, facilitates the transport and uptake of vitamin D metabolites (28–30), with genetic variations linked to altered vitamin D3 bioavailability and deficiency (31, 32). Recent studies also identified the hepatic vitamin D3 metabolite, 25OHD_3_-3-*O*-glucuronide, as a bona fide substrate of related intestinal ABC transporters (33). These observations support our earlier *in silico* study, demonstrating SDR42E1’s strong substrate affinity for vitamin D_3_ (9), underlining its potential role in vitamin D homeostasis and metabolism.

In alignment with the Hanahan and Weinberg framework (34), functional enrichment analysis indicates that *SDR42E1* disruption intersects multiple oncogenic hallmarks, particularly those related to colorectal cancer progression and chemotherapy response. The upregulation of *LRP1B*, *RGS5*, *STAT2*, and *HLA-A* in *SDR42E1*-depleted cells suggests enhanced apoptotic singling, tumor-immune crosstalk, and immune recognition—aligning with vitamin D’s immunomodulatory roles and potentially increasing chemotherapy sensitivity (35–37). Simultaneously, the downregulation of *WNT16*, cell cycle regulators such as *SLC7A5*, and histone genes, like *HIST2H3A* and *HIST1H2BJ*, points to disruptions in the Wnt signaling and chromatin remodeling, processes central to cancer cell proliferation and epigenetic regulation (5, 38–41). Additionally, transcriptional repression of DNA repair and senescence-related genes implies that *SDR42E1* may influence replicative stress and cell cycle arrest— mechanisms frequently dysregulated in malignancy (34). The concurrent downregulation of NADH dehydrogenase activity further implicates impaired redox homeostasis, consistent with vitamin D’s role in protecting against oxidative DNA damage (42). Together with observed reduction in HCT116 cell viability, these findings support a role for *SDR42E1* in modulating cell survival and senescence, both associated with poor cancer prognosis (43).

Conversely, overexpression of *SDR42E1* induced transcriptional changes consistent with stress response and apoptotic resistance. Notably, the upregulation of *HSPA5*, *CXCL8*, and *SERPINE1*, indicates activation of pro-survival and pro-metastatic pathways (44, 45). Elevated levels of SOX4, *DDIT3*, and *GADD45A*—genes implicated in stress response, DNA repair, and cancer stem cell maintenance—further suggest engagement of transcriptional mechanisms linked to cancer progression, therapy resistance, and recurrence (46, 47). Additionally, the downregulation of *CYP24A1*, a key enzyme in vitamin D catabolism and a biomarker of aggressive cancers, may sensitize cells to vitamin D-based therapies and conventional chemotherapy (48). Collectively, these findings highlight *SDR42E1* as a potential regulator of tumor biology and promising therapeutic target for improving cancer outcomes. These observations warrant further experimental validation through assays, such as Annexin V staining, apoptosis and cell cycle analysis—and mechanistic studies exploring the roles of chromatin remodeling and non-coding RNAs in mediating *SDR42E1*’s effects on vitamin D signaling and cancer-related pathways.

Consistent with transcriptomic data, proteomic profiling confirmed that *SDR42E1* knock-in influences key proteins alterations, showing downregulation of ALDOA, ALDOC, ABCC2, and FASN. These proteins regulate energy metabolism and lipid biosynthesis in liver and intestinal cell proliferation, potentially affecting cell viability (49). ALDOA, a glycolytic enzyme implicated in cancer (50), may link SDR42E1 to glucose metabolism and vitamin D regulation (51, 52). Given vitamin D deficiency’s association with muscle disorders and ALDOA elevation (53), further exploration of SDR42E1-ALDOA interactions is warranted.

Comparison of DEG in *SDR42E1*-depleted and -overexpressed models with GWAS catalog data (21) validated key regulators of vitamin D metabolism, including *CYP24A1*, *LIPE-AS1*, and *PCSK* (54–56). Additionally, *DHCR7* and *FDPS*, essential for synthesizing vitamin D precursors, were implicated (9, 57). Consistent with our previous findings, replicated alterations in *LDLR*, *LIPG*, *SLC7A5*, and *ABCA1* (16, 54) further support *SDR42E1*’s role in vitamin D absorption. These insights enhance our understanding of vitamin D homeostasis and *SDR42E1*’s to these intricate molecular processes.

Despite these valuable findings, certain limitations must be acknowledged. While HCT116 cells are relevant and well-characterized model for gene expression and molecular pathway analysis (58, 59), they may not fully reproduce the structural and functional complexity of vitamin D regulation in normal human intestinal or epithelial tissues. Their cancerous origin and limited differentiation potential could influence the generalizability of findings related to vitamin D absorption and metabolism. Nevertheless, their high endogenous expression of *SDR42E1* (16), along with key vitamin D-related genes, such as *ABCC2*, *CYP24A1*, and *CYP27B1* (60), supports their suitability for modeling *SDR42E1*-related molecular mechanisms in intestinal-like contexts. Moreover, although the CRISPR/Cas9 gene-editing approach enables precise genomic alterations, the potential for off-target effects remains a consideration. The lack of complementary functional assays—such as apoptosis, migration, or vitamin D rescue experiments— also constrains mechanistic interpretation. To address these limitations, further studies should include validation in primary human intestinal cells and *in vivo* models to better assess the physiological and therapeutic relevance of *SDR42E1* in vitamin D homeostasis and disease.

## 5 Conclusion

Overall, our study elucidates valuable insights into the role of *SDR42E1* in vitamin D metabolism and sterol processing, as well as its broader implications in cancer-related pathways. Through transcriptomic, proteomic, and functional analyses, we demonstrate that SDR42E1 disruption affects key regulators of vitamin D absorption, cell proliferation, and metabolic homeostasis. The findings advance our understanding of the molecular mechanisms underpinning vitamin D deficiency and highlight SDR42E1 as a potential molecular target. Importantly, this work opens avenues for exploring SDR42E1 modulation as a therapeutic strategy to enhance vitamin D bioavailability and potentially counteract tumorigenic processes associated with sterol dysregulation.

## Data Availability

The datasets presented in this study can be found in online repositories. The names of the repository/repositories and accession number(s) can be found in the article/**Supplementary Material**.

## References

[1] Mendoza A Takemoto Y Cruzado KT Masoud SS Nagata A Tantipanjaporn A. Controlled lipid beta-oxidation and carnitine biosynthesis by a vitamin D metabolite. Cell Chem Biol. (2022) 29:660–9 e12. doi: 10.1016/j.chembiol.2021.08.008 34506728

[2] Kiourtzidis M Kuhn J Brandsch C Stangl GI. Vitamin D status of mice deficient in scavenger receptor class B type 1, cluster determinant 36 and ATP-binding cassette proteins G5/G8. Nutrients. (2020) 12(8):E2295. doi: 10.3390/nu12082169 PMC7469065 32707802

[3] Zhang Y Li CN Jiang WD Wu P Liu Y Kuang SY. An emerging role of vitamin D(3) in amino acid absorption in different intestinal segments of on-growing grass carp (Ctenopharyngodon idella). Anim Nutr. (2022) 10:305–18. doi: 10.1016/j.aninu.2022.05.004 PMC9293741 35891684

[4] Ma R Gu Y Zhao S Sun J Groome LJ Wang Y. Expressions of vitamin D metabolic components VDBP, CYP2R1, CYP27B1, CYP24A1, and VDR in placentas from normal and preeclamptic pregnancies. Am J Physiol Endocrinol Metab. (2012) 303:E928–35. doi: 10.1152/ajpendo.00279.2012 PMC3469619 22871339

[5] Hendi NN Nemer G. Epigenetic regulation of vitamin D deficiency. Epigenomics. (2023) 15:653–5. doi: 10.2217/epi-2023-0246 37461377

[6] Persson B Kallberg Y Bray JE Bruford E Dellaporta SL Favia AD. (short-chain dehydrogenase/reductase and related enzymes) nomenclature initiative. Chem Biol Interact. (2009) 178:94–8. doi: 10.1016/j.cbi.2008.10.040 PMC2896744 19027726

[7] Bouhouche A Albaroudi N El Alaoui MA Askander O Habbadi Z El Hassani A. Identification of the novel SDR42E1 gene that affects steroid biosynthesis associated with the oculocutaneous genital syndrome. Exp Eye Res. (2021) 209:108671. doi: 10.1016/j.exer.2021.108671 34133966

[8] Chen S Liu W Yang C Li X Shen X Jiang D. Gonadotropin inhibitory hormone downregulates steroid hormone secretion and genes expressions in duck granulosa cells. Anim Reprod. (2021) 18:e20210036. doi: 10.1590/1984-3143-ar2021-0036 34306216 PMC8291778

[9] Hendi NN Nemer G. In silico characterization of the novel SDR42E1 as a potential vitamin D modulator. J Steroid Biochem Mol Biol. (2023) 238:106447. doi: 10.1016/j.jsbmb.2023.106447 38160768

[10] Loveland JL Lank DB Kupper C. Gene expression modification by an autosomal inversion associated with three male mating morphs. Front Genet. (2021) 12:641620. doi: 10.3389/fgene.2021.641620 34149796 PMC8213371

[11] Ogunmwonyi I Adebajo A Wilkinson JM. The genetic and epigenetic contributions to the development of nutritional rickets. Front Endocrinol (Lausanne). (2022) 13:1059034. doi: 10.3389/fendo.2022.1059034 36619587 PMC9815715

[12] Sinnott-Armstrong N Tanigawa Y Amar D Mars N Benner C Aguirre M. Genetics of 35 blood and urine biomarkers in the UK Biobank. Nat Genet. (2021) 53:185–94. doi: 10.1038/s41588-020-00757-z PMC7867639 33462484

[13] Revez JA Lin T Qiao Z Xue A Holtz Y Zhu Z. Genome-wide association study identifies 143 loci associated with 25 hydroxyvitamin D concentration. Nat Commun. (2020) 11:1647. doi: 10.1038/s41467-020-15421-7 32242144 PMC7118120

[14] Qiu S Zheng K Hu Y Liu G. Genetic correlation, causal relationship, and shared loci between vitamin D and COVID-19: A genome-wide cross-trait analysis. J Med Virol. (2023) 95:e28780. doi: 10.1002/jmv.28780 37212302

[15] Stiles AR Kozlitina J Thompson BM McDonald JG King KS Russell DW. Genetic, anatomic, and clinical determinants of human serum sterol and vitamin D levels. Proc Natl Acad Sci U S A. (2014) 111:E4006–14. doi: 10.1073/pnas.1413561111 PMC4183318 25201972

[16] Hendi NN Bengoechea-Alonso MT Ericsson J Nemer G. Functional characterization of the SDR42E1 reveals its role in vitamin D biosynthesis. Heliyon. (2024) 10:e36466. doi: 10.1016/j.heliyon.2024.e36466 39263177 PMC11387231

[17] Love MI Huber W Anders S. Moderated estimation of fold change and dispersion for RNA-seq data with DESeq2. Genome Biol. (2014) 15:550. doi: 10.1186/s13059-014-0550-8 25516281 PMC4302049

[18] Luo W Brouwer C. Pathview: an R/Bioconductor package for pathway-based data integration and visualization. Bioinformatics. (2013) 29:1830–1. doi: 10.1093/bioinformatics/btt285 PMC3702256 23740750

[19] Wu T Hu E Xu S Chen M Guo P Dai Z. clusterProfiler 4.0: A universal enrichment tool for interpreting omics data. Innovation (Camb). (2021) 2:100141. doi: 10.1016/j.xinn.2021.100141 34557778 PMC8454663

[20] Yoo M Shin J Kim J Ryall KA Lee K Lee S. DSigDB: drug signatures database for gene set analysis. Bioinformatics. (2015) 31:3069–71. doi: 10.1093/bioinformatics/btv313 PMC4668778 25990557

[21] Sollis E Mosaku A Abid A Buniello A Cerezo M Gil L. The NHGRI-EBI GWAS Catalog: knowledgebase and deposition resource. Nucleic Acids Res. (2023) 51:D977–D85. doi: 10.1093/nar/gkac1010 PMC9825413 36350656

[22] Dekker J Larson T Tzvetkov J Harvey VL Dowle A Hagan R. Spatial analysis of the ancient proteome of archeological teeth using mass spectrometry imaging. Rapid Commun Mass Spectrom. (2023) 37:e9486. doi: 10.1002/rcm.v37.8 36735645

[23] Schindelin J Arganda-Carreras I Frise E Kaynig V Longair M Pietzsch T. Fiji: an open-source platform for biological-image analysis. Nat Methods. (2012) 9:676–82. doi: 10.1038/nmeth.2019 PMC3855844 22743772

[24] Setchell KD Heubi JE Shah S Lavine JE Suskind D Al-Edreesi M. Genetic defects in bile acid conjugation cause fat-soluble vitamin deficiency. Gastroenterology. (2013) 144:945–55 e6; quiz e14-5. doi: 10.1053/j.gastro.2013.02.004 23415802 PMC4175397

[25] He XY Merz G Yang YZ Mehta P Schulz H Yang SY. Characterization and localization of human type10 17beta-hydroxysteroid dehydrogenase. Eur J Biochem. (2001) 268:4899–907. doi: 10.1046/j.0014-2956.2001.02421.2421.x 11559359

[26] Zmijewski MA. Nongenomic activities of vitamin D. Nutrients. (2022) 14(23):4953. doi: 10.3390/nu14235104 36501134 PMC9737885

[27] Beenken A Cerutti G Brasch J Guo Y Sheng Z Erdjument-Bromage H. Structures of LRP2 reveal a molecular machine for endocytosis. Cell. (2023) 186:821–36 e13. doi: 10.1016/j.cell.2023.01.016 36750096 PMC9993842

[28] Sodani K Patel A Kathawala RJ Chen ZS. Multidrug resistance associated proteins in multidrug resistance. Chin J Cancer. (2012) 31:58–72. doi: 10.5732/cjc.011.10329 22098952 PMC3777468

[29] Alam C Aufreiter S Georgiou CJ Hoque MT Finnell RH O’Connor DL. Upregulation of reduced folate carrier by vitamin D enhances brain folate uptake in mice lacking folate receptor alpha. Proc Natl Acad Sci U S A. (2019) 116:17531–40. doi: 10.1073/pnas.1907077116 PMC6717308 31405972

[30] Guo YX He LY Zhang M Wang F Liu F Peng WX. 1,25-Dihydroxyvitamin D3 regulates expression of LRP1 and RAGE *in vitro* and *in vivo*, enhancing Abeta1–40 brain-to-blood efflux and peripheral uptake transport. Neuroscience. (2016) 322:28–38. doi: 10.1016/j.neuroscience.2016.01.041 26820600

[31] Claro da Silva T Hiller C Gai Z Kullak-Ublick GA. Vitamin D3 transactivates the zinc and manganese transporter SLC30A10 via the Vitamin D receptor. J Steroid Biochem Mol Biol. (2016) 163:77–87. doi: 10.1016/j.jsbmb.2016.04.006 27107558

[32] Fan J Liu S Du Y Morrison J Shipman R Pang KS. Up-regulation of transporters and enzymes by the vitamin D receptor ligands, 1alpha,25-dihydroxyvitamin D3 and vitamin D analogs, in the Caco-2 cell monolayer. J Pharmacol Exp Ther. (2009) 330:389–402. doi: 10.1124/jpet.108.149815 19414624

[33] Gao C Liao MZ Han LW Thummel KE Mao Q. Hepatic transport of 25-hydroxyvitamin D(3) conjugates: A mechanism of 25-hydroxyvitamin D(3) delivery to the intestinal tract. Drug Metab Dispos. (2018) 46:581–91. doi: 10.1124/dmd.117.078881 PMC5896369 29467214

[34] Hanahan D Weinberg RA. Hallmarks of cancer: the next generation. Cell. (2011) 144:646–74. doi: 10.1016/j.cell.2011.02.013 21376230

[35] Kong P Wang X Gao YK Zhang DD Huang XF Song Y. RGS5 maintaining vascular homeostasis is altered by the tumor microenvironment. Biol Direct. (2023) 18:78. doi: 10.1186/s13062-023-00437-y 37986113 PMC10662775

[36] Xu C Li YM Sun B Zhong FJ Yang LY. ATE1 inhibits liver cancer progression through RGS5-mediated suppression of wnt/beta-catenin signaling. Mol Cancer Res. (2021) 19:1441–53. doi: 10.1158/1541-7786.MCR-21-0027 PMC9398136 34158395

[37] Anderson TS McCormick AL Daugherity EA Oladejo M Okpalanwaka IF Smith SL. Listeria-based vaccination against the pericyte antigen RGS5 elicits anti-vascular effects and colon cancer protection. Oncoimmunology. (2023) 12:2260620. doi: 10.1080/2162402X.2023.2260620 37781234 PMC10540654

[38] Sun Y Zhu D Chen F Qian M Wei H Chen W. SFRP2 augments WNT16B signaling to promote therapeutic resistance in the damaged tumor microenvironment. Oncogene. (2016) 35:4321–34. doi: 10.1038/onc.2015.494 PMC4994019 26751775

[39] Salhia B Kiefer J Ross JT Metapally R Martinez RA Johnson KN. Integrated genomic and epigenomic analysis of breast cancer brain metastasis. PloS One. (2014) 9:e85448. doi: 10.1371/journal.pone.0085448 24489661 PMC3906004

[40] Naldi M Andrisano V Fiori J Calonghi N Pagnotta E Parolin C. Histone proteins determined in a human colon cancer by high-performance liquid chromatography and mass spectrometry. J Chromatogr A. (2006) 1129:73–81. doi: 10.1016/j.chroma.2006.06.100 16887128

[41] Najumudeen AK Ceteci F Fey SK Hamm G Steven RT Hall H. The amino acid transporter SLC7A5 is required for efficient growth of KRAS-mutant colorectal cancer. Nat Genet. (2021) 53:16–26. doi: 10.1038/s41588-020-00753-3 33414552

[42] Dwivedi S Singh V Sen A Yadav D Agrawal R Kishore S. Vitamin D in disease prevention and cure-part I: an update on molecular mechanism and significance on human health. Indian J Clin Biochem. (2024). doi: 10.1007/s12291-024-01251-7 PMC12229305 40625600

[43] Liu S Dong Q Wang E. Rsf-1 overexpression correlates with poor prognosis and cell proliferation in colon cancer. Tumour Biol. (2012) 33:1485–91. doi: 10.1007/s13277-012-0399-y 22528946

[44] Li SJ Wei XH Zhan XM He JY Zeng YQ Tian XM. Adipocyte-derived leptin promotes PAI-1 -mediated breast cancer metastasis in a STAT3/miR-34a dependent manner. Cancers (Basel). (2020) 12(12):3741. doi: 10.3390/cancers12123864 33371368 PMC7767398

[45] Wang Q Ke S Liu Z Shao H He M Guo J. HSPA5 promotes the proliferation, metastasis and regulates ferroptosis of bladder cancer. Int J Mol Sci. (2023) 24(6):5475. doi: 10.3390/ijms24065144 36982218 PMC10048805

[46] Liu J Jiang G Mao P Zhang J Zhang L Liu L. Down-regulation of GADD45A enhances chemosensitivity in melanoma. Sci Rep. (2018) 8:4111. doi: 10.1038/s41598-018-22484-6 29515153 PMC5841426

[47] Berastegui N Ainciburu M Romero JP Garcia-Olloqui P Alfonso-Pierola A Philippe C. The transcription factor DDIT3 is a potential driver of dyserythropoiesis in myelodysplastic syndromes. Nat Commun. (2022) 13:7619. doi: 10.1038/s41467-022-35192-7 36494342 PMC9734135

[48] Kamiya S Nakamori Y Takasawa A Takasawa K Kyuno D Ono Y. Suppression of the vitamin D metabolizing enzyme CYP24A1 provides increased sensitivity to chemotherapeutic drugs in breast cancer. Oncol Rep. (2023) 49(5):85. doi: 10.3892/or.2023.8522 36928289

[49] Votava JA John SV Li Z Chen S Fan J Parks BW. Mining cholesterol genes from thousands of mouse livers identifies aldolase C as a regulator of cholesterol biosynthesis. J Lipid Res. (2024) 65:100525. doi: 10.1016/j.jlr.2024.100525 38417553 PMC10965479

[50] Pirovich DB Da’dara AA Skelly PJ. Multifunctional fructose 1,6-bisphosphate aldolase as a therapeutic target. Front Mol Biosci. (2021) 8:719678. doi: 10.3389/fmolb.2021.719678 34458323 PMC8385298

[51] Alvarez JA Ashraf A. Role of vitamin d in insulin secretion and insulin sensitivity for glucose homeostasis. Int J Endocrinol. (2010) 2010:351385. doi: 10.1155/2010/351385 20011094 PMC2778451

[52] Tang H Li D Li Y Zhang X Song Y Li X. Effects of vitamin D supplementation on glucose and insulin homeostasis and incident diabetes among nondiabetic adults: A meta-analysis of randomized controlled trials. Int J Endocrinol. (2018) 2018:7908764. doi: 10.1155/2018/7908764 30627160 PMC6304827

[53] Rasheed K Sethi P Bixby E. Severe vitamin d deficiency induced myopathy associated with rhabydomyolysis. N Am J Med Sci. (2013) 5:334–6. doi: 10.4103/1947-2714.112491 PMC3690793 23814767

[54] Warren T McAllister R Morgan A Rai TS McGilligan V Ennis M. The interdependency and co-regulation of the vitamin D and cholesterol metabolism. Cells. (2021) 10(8):2007. doi: 10.3390/cells10082007 34440777 PMC8392689

[55] Thunen A La Placa D Zhang Z Shively JE. Role of lncRNA LIPE-AS1 in adipogenesis. Adipocyte. (2022) 11:11–27. doi: 10.1080/21623945.2021.2013415 34957921 PMC8726699

[56] Sachan V Le Devehat M Roubtsova A Essalmani R Laurendeau JF Garcon D. PCSK7: A novel regulator of apolipoprotein B and a potential target against non-alcoholic fatty liver disease. Metabolism. (2024) 150:155736. doi: 10.1016/j.metabol.2023.155736 37967646

[57] Guarana WL Lima CAD Barbosa AD Crovella S Sandrin-Garcia P. Farnesyl diphosphate synthase gene associated with loss of bone mass density and alendronate treatment failure in patients with primary osteoporosis. Int J Mol Sci. (2024) 25(11):5623. doi: 10.3390/ijms25115623 38891810 PMC11172034

[58] Wierzbicka JM Binek A Ahrends T Nowacka JD Szydlowska A Turczyk L. Differential antitumor effects of vitamin D analogues on colorectal carcinoma in culture. Int J Oncol. (2015) 47:1084–96. doi: 10.3892/ijo.2015.3088 PMC4532196 26260259

[59] Hibler EA Jacobs ET Stone AD Sardo CL Galligan MA Jurutka PW. Associations between vitamin D-binding protein isotypes, circulating 25(OH)D levels, and vitamin D metabolite uptake in colon cancer cells. Cancer Prev Res (Phila). (2014) 7:426–34. doi: 10.1158/1940-6207.CAPR-13-0269 PMC3975660 24472850

[60] Jacobs ET Van Pelt C Forster RE Zaidi W Hibler EA Galligan MA. CYP24A1 and CYP27B1 polymorphisms modulate vitamin D metabolism in colon cancer cells. Cancer Res. (2013) 73:2563–73. doi: 10.1158/0008-5472.CAN-12-4134 PMC3630267 23423976

