# Supplementary material for: SDR42E1 modulates vitamin D absorption and cancer pathogenesis: insights from an in vitro model
Source: Front Endocrinol (Lausanne). 2025 Jul 18;16:1585859. doi: 10.3389/fendo.2025.1585859 (PMC12313483; doi:10.3389/fendo.2025.1585859)
Supplement: Supplementary file 1 [file DataSheet1.pdf]

Supplementary Material

Supplementary Tables

Table S1. List of the key resources, Related to Materials and methods.

Reagent or resource	Source	Identifier
**Antibodies**		
Anti-SDR42E1 rabbit monoclonal antibody	Invitrogen, USA	RRID: AB_2647060
Anti-beta-actin mouse monoclonal antibody	Sigma-Aldrich, USA	RRID: AB_476744
Peroxidase IgG fraction monoclonal anti-mouse IgG (H+L) secondary antibody HRP conjugate	Thermo Scientific, USA	RRID: AB_2533947
Anti-rabbit IgG light chain specific secondary antibody	Jackson ImmunoResearch, UK	RRID: AB_2339149
Mouse monoclonal anti-golgi 58K antibody	Sigma-Aldrich, USA	RRID: AB_477002
Recombinant rabbit HSP60-mitochondrial marker monoclonal antibody	Thermo Scientific, USA	HSPD1-2206R
Rabbit calreticulin-ER marker IgG polyclonal antibody	Thermo Scientific, USA	RRID: AB_2787722
Streptavidin Alexa Fluor 488 conjugate	Thermo Scientific, USA	RRID: AB_2315383
Goat Anti-Rabbit IgG Antibody (H+L), Biotinylated	Vector Laboratories, USA	RRID: AB_2313606
**Bacterial and virus strains**		
DH5α chemically competent *Escherichia coli*	Thermo Scientific, USA	EC0112
**Chemicals, peptides, and recombinant proteins**		
DMEM high D-Glucose	Sigma-Aldrich, USA	2609789
Fetal bovine serum (FBS)	Thermo Fisher Gibco, USA	CONS013064
Antibiotic-antimycotic	Thermo Fisher Gibco, USA	2585856
0.25% Trypsin-EDTA	Sigma-Aldrich, USA	T4049
Dulbecco's phosphate-buffered saline (DPBS)	Thermo Fisher Gibco, USA	2124947
Puromycin	Thermo Fisher Scientific, USA	A1113803
TRIzol reagent	Invitrogen, USA	15596026
High-capacity cDNA reverse transcriptase kit	Applied Biosystems, USA	4368814
PowerUp SYBR Green Master Mix	Applied Biosystems, USA	A25741
CellTiter-Glo® Luminescent Cell Viability Assay	Promega Corporation, USA	G755A
Lipofectamine 3000	Thermo Fisher Scientific, USA	2293283
Hoechst stain	Invitrogen, USA	33258
ProlongTM Antifade Histomount	Thermo Fisher Scientific, USA	P36930
**Critical commercial assays**		
Bicinchoninic acid (BCA) protein assay kit	Thermo Fisher Scientific, USA	23225
Protein A/G magnetic beads	Thermo Scientific, USA	88802

Novex NuPAGE 4-12% Bis-Tris SDS-polyacrylamide gel	Invitrogen, USA	NP0322BOX
Pierce ECL Western blotting substrate	Thermo Scientific, USA	32106
**Experimental models: Cell lines**		
Human colorectal carcinoma HCT116 cell line	ATCC, USA	RRID: CVCL_0291
**Oligonucleotides**		
Human oligonucleotides primer for the amplification of *SDR42E1*	Integrated DNA Technologies, USA	
**Recombinant DNA**		
HA-tag	Addgene, USA	RRID:Addgene_55182
**Software and algorithms**		
Snapgene	GSL Biotech LLC, USA	version 5.3.2
CLC Genomics Workbench	Qiagen, Germany	version 22.0.2
DESeq2	R package	version 1.34
pheatmap	R package	version 1.0.2
Pathview	R package	version 1.0.12
ClusterProfiler	R package	version 4.2.2
Limma	R package	version 3.56.2
GraphPad Prism	GraphPad Software, USA	version 9
Fiji-ImageJ	NIH, USA	version 1.53q
Progenesis QI	Waters, USA	version 2.2
Mascot Daemon	Matrix Science, UK	version 2.6.0
Nikon A1R confocal fluorescence microscope	Nikon Instruments, USA	AX / AX R with NSPARC
**Other**		
Phenylmethylsulfonyl fluoride (PMSF)	Thermo Scientific, USA	329-98-6
Dithiothreitol (DDT)	Thermo Scientific, USA	R0861
Pierce RIPA buffer	Thermo Scientific, USA	89901
PageBlue Protein Staining Solution	Thermo Scientific, USA	24620
Sanger sequencing service	Macrogen Inc., Korea	http://macrogen.com
Trans-Blot Turbo transfer system	BioRad, USA	1704150
NanoDrop 8000	Thermo Scientific, USA	ND-8000-GL
XCell SureLock electrophoresis system	Invitrogen, USA	ZA10001

Table S2. Differentially expressed transcripts in the *SDR42E1* knock-in HCT116 model, related to Figure 2.

Gene	Base Mean	log_2 FC	lfcSE	*P*-value	FDR	Gene Description
SOX4	6368.28	1.72	0.06	1.13E-176	1.79E-172	SRY-Box Transcription Factor 4: Regulates embryonic development and cell fate.
LRP1B	857.01	3.26	0.12	7.54E-171	5.97E-167	Low Density Lipoprotein Receptor-Related Protein 1B: Involved in lipid homeostasis and cellular processes.
SCG2	1606.52	2.25	0.08	4.88E-170	2.58E-166	Secretogranin II: Precursor of neuroendocrine signaling peptides.
PAM	12493.86	1.41	0.05	1.22E-156	4.83E-153	Peptidylglycine Alpha-Amidating Monooxygenase: Catalyzes neuropeptide biosynthesis.
WNT16	953.62	-2.76	0.11	9.81E-151	3.11E-147	Wnt Family Member 16: Regulates Wnt signaling, bone formation, and chemotherapy resistance.
RGS5	565.18	3.26	0.14	2.27E-121	5.98E-118	Regulator of G Protein Signaling 5: Modulates cardiovascular function.
TPBG	1518.58	2.00	0.09	4.52E-119	1.02E-115	Trophoblast Glycoprotein: A cell surface protein linked to development and cancer.
ADGRF1	2548.62	1.43	0.07	2.09E-101	4.14E-98	Adhesion G Protein-Coupled Receptor F1: Involved in cell adhesion and brain function.
PDLIM5	6044.26	1.19	0.06	7.31E-100	1.29E-96	PDZ and LIM Domain 5: Interacts with actin filaments, essential for muscle function.
ABCC2	7721.49	1.06	0.05	9.46E-98	1.50E-94	ATP Binding Cassette Subfamily C Member 2: Functions as a multidrug resistance transporter.
MAL2	2622.27	-1.48	0.07	2.18E-97	3.13E-94	Mal, T-Cell Differentiation Protein 2: Facilitates protein transport in epithelial cells.
MAN1A1	10455.35	1.25	0.06	1.75E-95	2.31E-92	Mannosidase Alpha Class 1A Member 1: Involved in glycoprotein maturation.
HIST2H3A	2797.47	-1.31	0.06	1.45E-92	1.77E-89	Histone Cluster 2 H3 Family Member A: Core histone protein involved in DNA packaging.
DST	19452.17	0.91	0.05	3.32E-86	3.76E-83	Dystonin: Maintains cellular integrity, particularly in the nervous system.
MTHFD2	5292.52	-1.04	0.05	5.68E-83	6.00E-80	Methylenetetrahydrofolate Dehydrogenase (NADP+ Dependent) 2: Functions in folate metabolism.
PSAT1	1928.96	-1.37	0.07	4.59E-82	4.55E-79	Phosphoserine Aminotransferase 1: Enzyme in serine biosynthesis.
PPFIBP1	3445.99	1.23	0.07	7.14E-80	6.65E-77	PTPRF Interacting Protein Binding Protein 1: Involved in synapse formation.
DKK1	944.75	-1.88	0.10	2.66E-76	2.34E-73	Dickkopf WNT Signaling Pathway Inhibitor 1: Wnt signaling antagonist, critical in development.

HIST1H2BJ	2494.22	-1.23	0.07	3.38E-76	2.81E-73	Histone Cluster 1 H2B Family Member J: Contributes to chromatin structure.
HIST2H2AA3	3444.34	-1.13	0.06	3.15E-70	2.50E-67	Histone Cluster 2 H2A Family Member A3: Plays a role in chromatin organization.
HIST1H2BO	3068.56	-1.08	0.06	1.62E-69	1.22E-66	Histone Cluster 1 H2B Family Member O: Core nucleosome component.
HIST2H2AA4	3473.74	-1.10	0.06	3.14E-68	2.26E-65	Histone Cluster 2 H2A Family Member A4: Regulates chromatin and transcription.
HIST1H2AH	2951.97	-1.10	0.06	7.64E-68	5.26E-65	Histone Cluster 1 H2A Family Member H: Involved in DNA packaging.
HIST1H2AG	5006.16	-1.00	0.06	9.04E-67	5.97E-64	Histone Cluster 1 H2A Family Member G: Essential for chromatin structure.
ITGA2	3450.14	1.33	0.08	4.47E-66	2.83E-63	Integrin Subunit Alpha 2: Mediates cell adhesion and signaling.
EHF	3273.33	1.05	0.06	4.58E-62	2.79E-59	ETS Homologous Factor: Regulates epithelial cell differentiation.
HIST1H1E	8716.34	-0.85	0.05	6.56E-62	3.85E-59	Histone Cluster 1 H1 Family Member E: Linker histone in chromatin structure.
HIST1H2BC	3602.35	-0.97	0.06	9.89E-61	5.59E-58	Histone Cluster 1 H2B Family Member C: Component of nucleosomes.
HIST1H1C	8412.10	-0.81	0.05	6.19E-60	3.38E-57	Histone Cluster 1 H1 Family Member C: Involved in chromatin compaction.
SLC7A5	2546.53	-1.07	0.07	1.48E-58	7.82E-56	Solute Carrier Family 7 Member 5: Amino acid transporter.
TFRC	9437.56	-0.85	0.05	2.82E-58	1.44E-55	Transferrin Receptor: Mediates iron uptake.
ZNF704	3058.81	1.02	0.06	5.90E-58	2.92E-55	Zinc Finger Protein 704: Transcriptional regulator.
INPP4B	2368.19	1.15	0.07	1.67E-56	8.03E-54	Inositol Polyphosphate-4-Phosphatase Type II B: Functions in phosphoinositide signaling.
RN7SL3	11749.33	-1.00	0.06	3.71E-56	1.73E-53	RNA, 7SL, Cytoplasmic 3: Part of the signal recognition particle.
FAM3C	3927.04	-0.97	0.06	5.14E-55	2.33E-52	Family With Sequence Similarity 3 Member C: Regulates cell differentiation.
HIST1H2AD	2315.02	-1.02	0.07	1.53E-53	6.71E-51	Histone Cluster 1 H2A Family Member D: Contributes to chromatin architecture.
HIST1H2BD	5241.72	-0.80	0.05	7.39E-51	3.16E-48	Histone Cluster 1 H2B Family Member D: DNA packaging protein.
HIST1H3B	3456.62	-0.89	0.06	1.23E-50	5.14E-48	Histone Cluster 1 H3 Family Member B: Nucleosome core component.
THSD4	881.71	1.86	0.13	2.04E-50	8.28E-48	Thrombospondin Type 1 Domain Containing 4: Regulates extracellular matrix.
EREG	16535.60	-0.76	0.05	2.38E-50	9.42E-48	Epiregulin: Growth factor involved in cell proliferation.
HIST3H2BB	1293.50	-1.16	0.08	1.51E-47	5.83E-45	Histone Cluster 3 H2B Family Member B: Regulates chromatin structure.
ASNS	2119.09	-1.02	0.07	2.08E-47	7.84E-45	Asparagine Synthetase: Catalyzes asparagine synthesis.
HIST2H2BF	4700.54	-0.97	0.07	3.52E-46	1.30E-43	Histone Cluster 2 H2B Family Member F: Involved in chromatin structure.
HIST2H4B	3280.40	-0.91	0.07	2.09E-45	7.52E-43	Histone Cluster 2 H4 Family Member B: Part of the nucleosome.
HIST1H2BK	3525.85	-0.92	0.07	2.71E-45	9.53E-43	Histone Cluster 1 H2B Family Member K: Essential for DNA packaging.
UBE2H	4229.01	0.83	0.06	3.11E-45	1.07E-42	Ubiquitin Conjugating Enzyme E2 H: Involved in protein ubiquitination.
HIST1H2AJ	2342.31	-0.93	0.07	3.70E-45	1.25E-42	Histone Cluster 1 H2A Family Member J: Nucleosome component.

DENND5B	2194.13	1.10	0.08	6.38E-44	2.11E-41	DENN Domain Containing 5B: Regulates intracellular trafficking.
HIST1H2AI	2481.64	-0.91	0.07	8.89E-44	2.87E-41	Histone Cluster 1 H2A Family Member I: Functions in chromatin remodeling.
INSIG1	1357.97	-1.08	0.08	4.83E-43	1.53E-40	Insulin Induced Gene 1: Regulates cholesterol homeostasis.
HIST2H4A	3322.75	-0.87	0.06	6.27E-43	1.95E-40	Histone Cluster 2 H4 Family Member A: Core nucleosome protein.
MEF2C	1145.58	1.19	0.09	1.28E-42	3.91E-40	Myocyte Enhancer Factor 2C: Regulates muscle differentiation.
HIST1H2AB	1207.15	-1.10	0.08	1.41E-42	4.21E-40	Histone Cluster 1 H2A Family Member B: Important for DNA organization.
ANXA3	2396.45	-0.90	0.07	2.12E-42	6.22E-40	Annexin A3: Involved in membrane trafficking.
HIST2H2BE	2527.37	-0.93	0.07	3.59E-42	1.03E-39	Histone Cluster 2 H2B Family Member E: Regulates chromatin function.
HIST1H2AM	2869.63	-1.01	0.08	7.24E-42	2.05E-39	Histone Cluster 1 H2A Family Member M: DNA packaging protein.
HIST1H3J	1317.66	-1.15	0.09	1.19E-40	3.30E-38	Histone Cluster 1 H3 Family Member J: Crucial for DNA compaction.
HIST1H3H	2825.81	-0.90	0.07	1.49E-40	4.06E-38	Histone Cluster 1 H3 Family Member H: Involved in gene regulation.
ADGRG6	3678.67	0.82	0.06	2.11E-40	5.66E-38	Adhesion G Protein-Coupled Receptor G6: Functions in nervous and cardiovascular systems.
RRM2	2775.74	-1.07	0.08	4.49E-40	1.18E-37	Ribonucleotide Reductase Regulatory Subunit M2: Key in DNA synthesis.
IARS	9732.62	-0.69	0.05	6.04E-40	1.57E-37	Isoleucyl-tRNA Synthetase: Catalyzes isoleucine-tRNA attachment.
IDI1	1573.26	-0.98	0.08	1.96E-39	5.00E-37	Isopentenyl-Diphosphate Delta Isomerase 1: Essential in cholesterol biosynthesis.
H2AFZ	7600.28	-0.79	0.06	2.17E-39	5.41E-37	H2A.Z Variant Histone 1: Regulates chromatin dynamics.
EZR	11537.52	-0.65	0.05	2.18E-39	5.41E-37	Ezrin: Links cytoskeleton to the membrane.
PCNA	3423.72	-0.88	0.07	2.70E-39	6.58E-37	Proliferating Cell Nuclear Antigen: Supports DNA replication.
HIST1H3D	2101.89	-0.94	0.07	3.56E-39	8.54E-37	Histone Cluster 1 H3 Family Member D: Regulates chromatin function.
ATAD2	4220.03	-0.90	0.07	5.94E-39	1.40E-36	ATPase Family AAA Domain Containing 2: Involved in chromatin remodeling.
TFDP2	1900.00	0.98	0.08	3.24E-38	7.56E-36	Transcription Factor Dp-2: Regulates cell cycle progression.
FASN	6940.02	-0.77	0.06	6.44E-38	1.48E-35	Fatty Acid Synthase: Catalyzes fatty acid synthesis.
PLAT	435.46	1.73	0.14	8.04E-38	1.82E-35	Plasminogen Activator, Tissue Type: Involved in blood clot breakdown.
DDX21	5910.10	-0.86	0.07	1.68E-37	3.74E-35	DEAD-Box Helicase 21: Functions in RNA processing.
HIST1H2AC	3222.30	-0.86	0.07	6.21E-37	1.37E-34	Histone Cluster 1 H2A Family Member C: Contributes to DNA packaging.
ZFP36L1	2551.50	0.81	0.07	1.03E-36	2.23E-34	Zinc Finger Protein 36 Like 1: Regulates mRNA stability.
MAD2L1	2212.00	-0.86	0.07	6.29E-36	1.35E-33	Mitotic Arrest Deficient 2 Like 1: Ensures chromosome segregation.
SLC7A11	1717.97	-1.10	0.09	1.30E-34	2.74E-32	Solute Carrier Family 7 Member 11: Amino acid exchanger.
EIF2S2	2930.38	-0.76	0.06	1.45E-34	3.02E-32	Eukaryotic Translation Initiation Factor 2 Subunit Beta: Regulates protein synthesis.
HIST1H4B	1427.00	-0.98	0.08	4.93E-34	1.01E-31	Histone Cluster 1 H4 Family Member B: Essential for chromatin structure.

MCM4	5163.43	-0.83	0.07	5.24E-33	1.06E-30	Minichromosome Maintenance Complex Component 4: Key in DNA replication initiation.
LRRC59	3369.86	-0.87	0.07	6.48E-33	1.30E-30	Leucine Rich Repeat Containing 59: Involved in protein trafficking.
RALGAPA1	1008.17	-1.05	0.09	7.85E-33	1.55E-30	Ral GTPase Activating Protein Catalytic Alpha Subunit 1: Regulates intracellular signaling.
NUP58	2821.96	-0.78	0.07	1.18E-32	2.30E-30	Nucleoporin 58: Nuclear pore complex component.
TP53INP1	1184.37	1.10	0.09	2.18E-32	4.20E-30	Tumor Protein P53 Inducible Nuclear Protein 1: Modulates p53 activity.
PTP4A1	3986.33	-0.82	0.07	4.76E-32	9.08E-30	Protein Tyrosine Phosphatase Type IVA Member 1: Controls cell proliferation.
TMEM200A	734.05	1.27	0.11	5.15E-32	9.70E-30	Transmembrane Protein 200A: Potential role in signaling.
CTNNAL1	1945.12	-0.82	0.07	6.33E-32	1.18E-29	Catenin Alpha Like 1: Involved in cell adhesion.
ID3	1857.83	0.98	0.08	6.44E-32	1.19E-29	Inhibitor of DNA Binding 3: Regulates differentiation.
TUBA1C	5421.86	-0.70	0.06	8.70E-32	1.58E-29	Tubulin Alpha 1c: Essential for microtubule formation.
HIST3H2A	996.25	-1.04	0.09	9.50E-32	1.71E-29	Histone Cluster 3 H2A: Contributes to chromatin regulation.
HSPA9	9621.10	-0.60	0.05	1.10E-31	1.96E-29	Heat Shock Protein Family A (Hsp70) Member 9: Assists protein folding.
TRIB1	8197.35	0.56	0.05	2.40E-31	4.22E-29	Tribbles Pseudokinase 1: Modulates apoptosis.
PERP	4378.57	0.70	0.06	4.54E-31	7.91E-29	PERP, TP53 Apoptosis Effector: Mediates p53-dependent apoptosis.
MPP7	5725.63	0.59	0.05	5.78E-31	9.95E-29	Membrane Palmitoylated Protein 7: Maintains cell polarity.
CYCS	4234.61	-0.81	0.07	4.58E-30	7.80E-28	Cytochrome C, Somatic: Functions in apoptosis.
EPS8	2900.28	0.68	0.06	6.16E-30	1.04E-27	EGFR Pathway Substrate 8: Involved in actin remodeling.
AREG	2768.71	-0.94	0.08	8.94E-30	1.49E-27	Amphiregulin: Regulates cell proliferation.
GARS	2228.58	-0.75	0.07	9.35E-30	1.54E-27	Glycyl-tRNA Synthetase: Catalyzes glycine-tRNA attachment.
LOC107985779	154.23	2.84	0.26	2.21E-29	3.61E-27	No approved symbol: Hypothetical protein with unknown function.

DEGs linked to *SDR42E1* show FDR values < 5E−27, calculated by the Benjamini-Hochberg method in DESeq2. Abbreviations: lfcSE, standard error of the $\log_2$ Fold Change (FC) estimate; FDR, false discovery rate represents adjusted P-value using the Benjamini Hochberg in DESeq2.

Table S3. Differentially expressed proteins in the SDR42E1 knock-in HCT116 model: related to Figure 3.

Protein	log_2 FC	Average Expression	t-test	*P*-value	q-value	Protein description
ALDOA	-1.32	12.42	-9.73	4.57E-06	0.0060	Aldolase, Fructose-Bisphosphate A: Glycolytic enzyme and tumor promoter.
HIST1H4A	2.79	10.03	8.77	1.07E-05	0.0071	Histone H4: Core nucleosome component essential for chromatin structure.
HIST2H3A	2.48	9.41	6.71	8.87E-05	0.0390	Histone H3.2: H3 variant involved in nucleosome formation and gene regulation.
FASN	-0.69	13.81	-6.43	0.0001	0.0405	Fatty Acid Synthase: Catalyzes long-chain fatty acid synthesis.
ALDOC	-1.23	10.21	-5.74	0.0003	0.0749	Aldolase: Fructose-Bisphosphate C: Glycolytic enzyme mainly in the brain.
HIST1H2BK	2.29	9.24	5.25	0.0005	0.1174	Histone H2B: Nucleosome protein involved in chromatin organization.
ABHD10	1.49	8.05	5.05	0.0007	0.1312	Abhydrolase Domain Containing 10: Thioesterase enzyme in lipid metabolism.
AKAP12	-0.82	11.01	-4.82	0.0010	0.1578	A Kinase (PRKA) Anchor Protein 12: Scaffolding protein for PKA signaling.
RPL11	0.97	9.26	4.72	0.0011	0.1601	Ribosomal Protein L11: 60S ribosomal subunit component for translation.
RPL35	1.10	8.10	4.59	0.0013	0.1736	Ribosomal Protein L35: 60S ribosomal subunit protein aiding translation.
MAP4	-0.87	8.93	-4.49	0.0015	0.1834	Microtubule-Associated Protein 4: Stabilizes microtubules for cell structure.
CFL1	1.01	11.74	4.34	0.0019	0.2068	Cofilin 1: Actin-binding protein regulating cytoskeleton dynamics.
ACSL5	-1.32	7.88	-4.22	0.0023	0.2296	Acyl-CoA Synthetase Long 5: Converts long-chain fatty acids to acyl-CoA.
PDLIM5	1.36	7.11	4.15	0.0025	0.2359	PDZ And LIM Domain 5: Cytoskeletal protein linked to muscle function.
GSTP1	0.87	11.19	4.02	0.0030	0.2653	Glutathione S-Transferase Pi 1: Detoxification enzyme conjugating glutathione.
RPS12	1.38	7.44	3.95	0.0034	0.2730	Ribosomal Protein S12: 40S ribosomal subunit component for translation.
H2AFY	2.77	5.10	3.92	0.0035	0.2730	H2A Histone Family: Member Y: H2A variant involved in chromatin remodeling.
PEBP1	0.97	9.10	3.83	0.0040	0.2871	Phosphatidylethanolamine Binding Protein 1: Binds phospholipids: modulating signaling.
PNP	1.49	5.89	3.82	0.0041	0.2871	Purine Nucleoside Phosphorylase: Enzyme in purine salvage metabolism.
RPS24	1.40	7.86	3.77	0.0044	0.2932	Ribosomal Protein S24: 40S ribosomal subunit component in translation.
EIF4B	-0.87	7.86	-3.68	0.0051	0.3033	Eukaryotic Translation Initiation Factor 4B: Aids mRNA recruitment to ribosomes.
LARP4	-1.17	8.28	-3.62	0.0056	0.3033	La Ribonucleoprotein Domain 4: RNA-binding protein regulating translation.
PFN1	0.70	11.60	3.61	0.0057	0.3033	Profilin 1: Regulates actin polymerization and cell movement.
APRT	0.92	8.07	3.57	0.0060	0.3033	Adenine Phosphoribosyltransferase: Converts adenine to AMP in purine salvage.
ACTN1	1.01	7.41	3.56	0.0061	0.3033	Actinin Alpha 1: Crosslinks actin filaments in the cytoskeleton.
CYB5R3	0.80	7.88	3.55	0.0062	0.3033	Cytochrome B5 Reductase 3: Electron transport enzyme for metabolism.
RPS4X	0.63	10.13	3.52	0.0065	0.3033	Ribosomal Protein S4: X-Linked: 40S ribosomal subunit component.
YWHAE	0.53	10.83	3.52	0.0066	0.3033	Tyrosine 3-Monooxygenase/Tryptophan 5-Monooxygenase Activation Protein Epsilon: Scaffolding protein for signaling pathways.
USP10	-1.01	7.02	-3.51	0.0067	0.3033	Ubiquitin Specific Peptidase 10: Deubiquitinating enzyme regulating protein turnover.
PSMA2	1.08	7.65	3.45	0.0073	0.3056	Proteasome Subunit Alpha Type-2: 20S proteasome core component.

MTAP	0.72	9.79	3.44	0.0074	0.3056	Methylthioadenosine Phosphorylase: Enzyme in methionine salvage.
ATP1A1	-0.59	10.66	-3.44	0.0074	0.3056	ATPase Na+/K+ Transporting Alpha 1: Maintains ion balance via Na+/K+ ATPase.
RPLP1	0.98	9.42	3.41	0.0078	0.3056	Ribosomal Protein: Large: P1: 60S ribosomal subunit protein for translation.
RPS26	0.96	7.30	3.38	0.0081	0.3056	Ribosomal Protein S26: 40S ribosomal subunit component.
RPS11	0.60	9.41	3.38	0.0082	0.3056	Ribosomal Protein S11: 40S ribosomal subunit component.
SNRPD3	1.08	7.42	3.35	0.0085	0.3056	Small Nuclear Ribonucleoprotein D3: Spliceosome protein for pre-mRNA splicing.
RPL12	0.62	10.19	3.35	0.0086	0.3056	Ribosomal Protein L12: 60S ribosomal subunit component.
HPRT1	1.37	6.70	3.28	0.0096	0.3213	Hypoxanthine Phosphoribosyltransferase 1: Converts hypoxanthine to IMP in purine salvage.
SRP14	0.92	7.43	3.28	0.0096	0.3213	Signal Recognition Particle 14: Directs proteins to the ER.
YWHAB	0.65	8.31	3.26	0.0099	0.3213	Tyrosine 3-Monooxygenase/Tryptophan 5-Monooxygenase Activation Protein Beta: Regulates signaling and cell cycle.
HSD17B10	1.12	6.32	3.25	0.0100	0.3213	Hydroxysteroid 17-Beta Dehydrogenase 10: Enzyme in steroid metabolism.
USP5	-0.54	9.50	-3.22	0.0105	0.3213	Ubiquitin Specific Peptidase 5: Deubiquitinating enzyme.
CAD	-0.67	8.62	-3.21	0.0107	0.3213	Carbamoyl-Phosphate Synthetase 2: Aspartate Transcarbamylase, and Dihydroorotase: Pyrimidine biosynthesis enzyme.
PSMA3	1.19	7.53	3.20	0.0109	0.3213	Proteasome Subunit Alpha Type-3: 20S proteasome core component.
ATP5O	0.95	7.88	3.19	0.0111	0.3213	ATP Synthase, H+ Transporting, F1 Complex O: Mitochondrial ATP production enzyme.
PHB	0.46	11.37	3.18	0.0112	0.3213	Prohibitin: Regulates mitochondria: cell cycle: and apoptosis.
CLTC	-0.46	11.97	-3.17	0.0114	0.3213	Clathrin Heavy Chain: Involved in intracellular vesicle transport.
RPL23	0.63	9.40	3.15	0.0118	0.3232	Ribosomal Protein L23: 60S ribosomal subunit protein.
PGK1	-0.46	12.08	-3.13	0.0121	0.3264	Phosphoglycerate Kinase 1: Glycolysis enzyme converting 1,3-bisphosphoglycerate.
HMGB1	0.73	10.38	3.11	0.0126	0.3299	High Mobility Group Box 1: DNA-binding protein regulating transcription.
PSMA7	0.68	8.06	3.08	0.0131	0.3299	Proteasome Subunit Alpha Type-7: 20S proteasome core component.
PDIA3	-0.40	12.26	-3.08	0.0132	0.3299	Protein Disulfide Isomerase A3: Assists in protein disulfide bond formation.
FABP5	0.65	9.34	3.07	0.0134	0.3299	Fatty Acid Binding Protein 5: Transports intracellular fatty acids.
LMNB1	0.64	8.95	3.06	0.0136	0.3299	Lamin B1: Nuclear lamina component regulating transcription.
EIF6	1.05	7.74	3.04	0.0139	0.3299	Eukaryotic Translation Initiation Factor 6: Prevents premature 80S ribosome assembly.
VARS	-0.69	10.19	-3.01	0.0147	0.3299	Valyl-tRNA Synthetase: Attaches valine to tRNA in translation.
SLC2A1	-0.92	7.14	-3.00	0.0149	0.3299	Solute Carrier Family 2 Member 1: Glucose transporter.
SRSF3	0.78	9.23	3.00	0.0149	0.3299	Serine and Arginine Rich Splicing Factor 3: Involved in mRNA splicing and export.
PPIA	0.60	12.80	3.00	0.0150	0.3299	Peptidylprolyl Isomerase A: Catalyzes isomerization of proline residues in protein folding.

ANXA5	0.50	10.63	2.97	0.0156	0.3299	Annexin A5: A calcium-dependent phospholipid-binding protein in membrane events.
CDCP1	-2.06	4.17	-2.97	0.0157	0.3299	CUB Domain Containing Protein 1: Implicated in cell adhesion and signaling.
HNRNPH3	0.56	8.51	2.97	0.0158	0.3299	Heterogeneous Nuclear Ribonucleoprotein H3: Involved in mRNA processing & transport.
RPL9	0.73	9.17	2.96	0.0161	0.3299	Ribosomal Protein L9: A 60S ribosomal subunit component essential for protein synthesis.
PHB2	0.47	10.89	2.96	0.0161	0.3299	Prohibitin 2: Functions in mitochondrial protein folding and maintenance.
RCN1	-1.05	6.23	-2.95	0.0162	0.3299	Reticulocalbin 1: Involved in calcium binding in the endoplasmic reticulum.
FKBP4	-0.45	10.10	-2.93	0.0168	0.3365	FK506 Binding Protein 4: A chaperone that binds FK506 immunosuppressive drugs.
RPL17	0.64	9.31	2.92	0.0171	0.3365	Ribosomal Protein L17: A 60S ribosomal component essential for protein synthesis.
RPL30	0.68	8.22	2.89	0.0179	0.3428	Ribosomal Protein L30: A 60S ribosomal component essential for protein synthesis.
DDOST	0.69	9.31	2.88	0.0182	0.3428	Dolichyl-Diphosphooligosaccharide-Protein Glycosyltransferase: Involved in early protein glycosylation.
KIF5B	-0.78	7.84	-2.88	0.0182	0.3428	Kinesin Family Member 5B: A motor protein in vesicle transport along microtubules.
PSMD3	-0.60	8.50	-2.87	0.0185	0.3437	Proteasome 26S Subunit: Non-ATPase 3: Part of 26S proteasome for protein degradation.
ARPC4	0.81	7.30	2.85	0.0192	0.3486	Actin Related Protein 2/3 Complex Subunit 4: A component of Arp2/3 complex in actin nucleation.
NUDT21	3.78	4.03	2.84	0.0195	0.3486	Nudix Hydrolase 21: Involved in mRNA processing and polyadenylation.
GNAI2	0.85	7.15	2.84	0.0195	0.3486	G Protein Subunit Alpha i2: A heterotrimeric G-protein in inhibitory signaling.
TOMM22	1.67	7.93	2.83	0.0199	0.3504	Translocase Of Outer Mitochondrial Membrane 22: Imports proteins into mitochondria.
BTF3	0.93	7.55	2.81	0.0205	0.3561	Basic Transcription Factor 3: Regulates transcription via RNA polymerase II.
RPS16	0.84	8.27	2.78	0.0216	0.3651	Ribosomal Protein S16: A 40S ribosomal component essential for protein synthesis.
RPS13	0.73	9.12	2.77	0.0219	0.3651	Ribosomal Protein S13: A 40S ribosomal component essential for protein synthesis.
EIF5A	0.91	9.19	2.76	0.0220	0.3651	Eukaryotic Translation Initiation Factor 5A: Functions in translation initiation and cell proliferation.
ACTC1	0.60	10.89	2.76	0.0221	0.3651	Actin: Alpha Cardiac Muscle 1: A structural protein in muscle contraction.
SLC16A3	-1.82	5.35	-2.74	0.0229	0.3678	Solute Carrier Family 16 Member 3: Also known as MCT4: involved in lactate transport.
ASPH	-0.90	7.17	-2.74	0.0229	0.3678	Aspartate Beta-Hydroxylase: Hydroxylates aspartate residues in proteins.
PFKP	-0.79	10.03	-2.73	0.0231	0.3678	Phosphofructokinase: Platelet: A key regulatory enzyme in glycolysis.
RPL10A	0.50	9.58	2.72	0.0235	0.3689	Ribosomal Protein L10a: A 60S ribosomal component essential for protein synthesis.
PARK7	0.96	9.43	2.71	0.0241	0.3737	Parkinsonism Associated Deglycase: Protects cells from oxidative stress.
RPL22L1	1.82	7.30	2.70	0.0244	0.3742	Ribosomal Protein L22 Like 1: A 60S ribosomal component vital for protein synthesis.
SARS	-0.61	8.25	-2.69	0.0247	0.3742	Seryl-tRNA Synthetase: Attaches serine to its tRNA during protein synthesis.

YWHAG	0.62	9.24	2.65	0.0265	0.3974	14-3-3 Protein Gamma: Involved in phosphoserine-dependent signaling.
CDV3	-1.34	5.72	-2.63	0.0274	0.4041	CDV3 Homolog: Putatively involved in cell signaling and growth.
PSMA1	0.76	8.40	2.63	0.0276	0.4041	Proteasome Subunit Alpha 1: Part of the 20S proteasome core for protein degradation.
CTSD	0.75	7.01	2.61	0.0282	0.4041	Cathepsin D: A lysosomal enzyme in protein degradation.
MYL6	0.60	7.75	2.60	0.0287	0.4041	Myosin Light Chain 6: A myosin component in muscle contraction.
PSMB3	0.95	6.52	2.60	0.0288	0.4041	Proteasome Subunit Beta 3: Part of the 20S proteasome core for protein degradation.
SLC9A3R1	-0.54	8.22	-2.59	0.0290	0.4041	Solute Carrier Family 9A3 Regulator 1, Also known as NHERF1: regulates ion transport.
RAB11B	0.77	8.21	2.59	0.0291	0.4041	RAB11B: Member RAS Oncogene Family: Involved in endosomal recycling.
EIF5B	-0.70	7.33	-2.57	0.0300	0.4125	Eukaryotic Translation Initiation Factor 5B: Promotes 80S ribosome assembly.
RPL18A	0.60	9.05	2.55	0.0312	0.4199	Ribosomal Protein L18a: A 60S ribosomal component essential for protein synthesis.
CHORDC1	-0.83	7.85	-2.55	0.0313	0.4199	Cysteine and Histidine Rich Domain Containing 1: Involved in protein folding and stress response.
DPYSL2	-0.88	6.85	-2.55	0.0315	0.4199	Dihydropyrimidinase Like 2: Functions in neuronal development and axonal guidance.
NUP155	-1.16	5.36	-2.53	0.0322	0.4236	Nucleoporin 155: A nuclear pore complex component in nucleocytoplasmic transport.
PRDX1	0.53	11.91	2.51	0.0333	0.4236	Peroxiredoxin 1: An antioxidant enzyme that reduces peroxides.
LMNB2	0.56	8.63	2.51	0.0333	0.4236	Lamin B2: A nuclear lamina component involved in structural integrity.
RRBP1	-0.73	7.23	-2.50	0.0337	0.4236	Ribosome Binding Protein 1: Involved in ribosome binding to the ER.
EIF4G1	-0.38	9.83	-2.50	0.0338	0.4236	Eukaryotic Translation Initiation Factor 4 Gamma 1: Aids in translation initiation by binding mRNA.
RPL15	-0.42	9.79	-2.50	0.0341	0.4236	Ribosomal Protein L15: A 60S ribosomal component essential for protein synthesis.
PGAM1	0.58	11.00	2.49	0.0344	0.4236	Phosphoglycerate Mutase 1: Converts 3- to 2-phosphoglycerate in glycolysis.
ITGA6	-0.75	6.55	-2.49	0.0346	0.4236	Integrin Subunit Alpha 6: A cell surface receptor in adhesion and signaling.
U2SURP	-2.00	4.60	-2.49	0.0347	0.4236	U2 Small Nuclear RNA Auxiliary Factor 1: Functions in RNA splicing.
TCERG1	-0.89	6.39	-2.47	0.0357	0.4290	Transcription Elongation Regulator 1: Regulates transcription elongation and RNA processing.
UNC45A	1.25	4.93	2.46	0.0361	0.4290	Unc-45 Myosin Chaperone A: Involved in myosin folding and muscle assembly.
EIF4H	0.49	8.82	2.46	0.0361	0.4290	Eukaryotic Translation Initiation Factor 4H: Enhances EIF4A activity in RNA unwinding.
CAP1	-0.53	9.32	-2.46	0.0364	0.4290	Cyclase Associated Actin Cytoskeleton Regulatory Protein 1: Regulates actin filament dynamics.
SNRPA1	0.75	7.66	2.45	0.0371	0.4292	Small Nuclear Ribonucleoprotein Polypeptide A': A spliceosome component in mRNA splicing.

AIFM1	0.50	9.33	2.45	0.0371	0.4292	Apoptosis Inducing Factor Mitochondria Associated 1: Induces apoptosis & maintains mitochondria.
ARHGDIA	0.40	11.06	2.44	0.0374	0.4292	Rho GDP Dissociation Inhibitor Alpha: Regulates Rho GTPases by inhibiting GDP dissociation.
TPI1	0.48	12.88	2.43	0.0379	0.4309	Triosephosphate Isomerase 1: Catalyzes glycolytic interconversion of DHAP and G3P.
RANBP2	-0.95	6.66	-2.42	0.0386	0.4309	RAN Binding Protein 2: A nucleoporin involved in nucleocytoplasmic transport.
DSG2	-0.75	7.24	-2.41	0.0391	0.4309	Desmoglein 2: A desmosomal protein in epithelial cell adhesion.
PSMA4	1.09	7.00	2.41	0.0395	0.4309	Proteasome Subunit Alpha 4: Part of the 20S proteasome core for protein degradation.
DIAPH1	-0.95	6.43	-2.41	0.0395	0.4309	Diaphanous Related Formin 1: Regulates actin polymerization and morphology.
SNRPD1	-2.47	6.29	-2.40	0.0398	0.4309	Small Nuclear Ribonucleoprotein D1 Polypeptide: A spliceosome component in mRNA splicing.
HYOU1	-0.75	9.36	-2.40	0.0398	0.4309	Hypoxia Up-Regulated 1: A chaperone in protein folding under hypoxia.
LMNA	0.39	12.58	2.39	0.0404	0.4317	Lamin A/C: A nuclear lamina component for structural integrity.
UPF1	-0.84	7.15	-2.39	0.0406	0.4317	UPF1 Regulator Of Nonsense Transcripts Homolog: Functions in nonsense-mediated mRNA decay.
PDP1	-0.88	5.66	-2.38	0.0410	0.4329	Pyruvate Dehydrogenase Phosphatase 1: Regulates the pyruvate dehydrogenase complex.
ACLY	-0.42	10.42	-2.37	0.0419	0.4390	ATP Citrate Lyase: Converts citrate to acetyl-CoA for lipid biosynthesis.
CD109	-1.89	3.52	-2.36	0.0429	0.4462	CD109 Molecule: A GPI-anchored protein in cell growth regulation.
RPA1	-0.86	6.52	-2.34	0.0437	0.4477	Replication Protein A1: Functions in DNA replication and repair.
PFAS	-0.84	6.44	-2.34	0.0438	0.4477	Phosphoribosylformylglycinamidine Synthase: Involved in purine biosynthesis.
GPI	-0.33	11.31	-2.32	0.0452	0.4569	Glucose-6-Phosphate Isomerase: Catalyzes glucose-6-phosphate to fructose-6-phosphate.
RPS18	0.57	9.48	2.32	0.0453	0.4569	Ribosomal Protein S18: A 40S ribosomal component essential for protein synthesis.
EBP	-0.81	6.40	-2.31	0.0461	0.4608	Emopamil Binding Protein: Involved in cholesterol biosynthesis.
PRDX6	0.42	10.77	2.30	0.0470	0.4665	Peroxiredoxin 6: An antioxidant enzyme in peroxide reduction and lipid metabolism.
ZYX	-1.10	5.94	-2.29	0.0478	0.4702	Zyxin: Regulates actin filament assembly and cell adhesion.
RPL24	0.44	9.49	2.29	0.0481	0.4702	Ribosomal Protein L24: A 60S ribosomal component essential for protein synthesis.
NASP	-0.61	9.07	-2.28	0.0484	0.4702	Nuclear Autoantigenic Sperm Protein: Functions in histone transport and assembly.
EEF1A1	-0.30	14.28	-2.28	0.0489	0.4706	Eukaryotic Translation Elongation Factor 1 Alpha 1: Aids elongation in protein synthesis.
IDH2	-2.99	2.16	-2.27	0.0496	0.4706	Isocitrate Dehydrogenase 2: Converts isocitrate to alpha-ketoglutarate in the TCA cycle.
PSMB2	0.83	6.46	2.27	0.0497	0.4706	Proteasome Subunit Beta 2: Part of the 20S proteasome core for protein degradation.
COPA	-0.51	8.35	-2.26	0.0499	0.4706	Coatomer Protein Complex Subunit Alpha: Transports proteins from ER to Golgi.

Differentially expressed proteins identified from the proteomic analysis of the *SDR42E1* knockin HCT116 model with *P*-values calculated by the Benjamini-Hochberg method in Limma.

Table S4 Differentially expressed genes in the wild-type overexpression *SDR42E1* HCT116 model, related to Figure 4.

**Gene**	**Base Mean**	**log_2 FC**	**lfcSE**	***P*-value**	**FDR**	**Gene Description**
SDR42E1	105440.61	7.90	0.15	< 2.2E−308	< 2.2E−308	Short Chain Dehydrogenase/Reductase 42E 1: Involved in steroid metabolism.
DDIT3	634.28	3.11	0.16	8.72E-87	6.12E-83	DNA Damage Inducible Transcript 3: Regulates ER stress response and apoptosis.
CXCL8	369.78	5.31	0.28	8.76E-79	4.10E-75	C-X-C Motif Chemokine Ligand 8: Mediates inflammatory response.
ATF3	528.08	2.42	0.15	1.41E-56	4.95E-53	Activating Transcription Factor 3: Responds to stress & regulates gene expression.
GADD45A	621.40	1.72	0.13	2.69E-39	7.56E-36	Growth Arrest & DNA Damage Inducible Alpha: Involved in DNA damage stress signaling.
HSPA5	5961.11	1.22	0.10	4.20E-33	9.85E-30	Heat Shock Protein A (Hsp70) 5: Facilitates protein folding & ER stress response.
GALNT5	973.52	1.26	0.12	1.27E-25	2.55E-22	Polypeptide N-Acetylgalactosaminyltransferase 5: Catalyzes O-linked glycosylation.
SERPINE1	427.59	1.77	0.18	1.31E-23	2.30E-20	Serpin E1: Regulates fibrinolysis as a serine protease inhibitor.
ABCC2	3347.71	-1.09	0.11	1.96E-23	3.05E-20	ATP Binding Cassette Subfamily C2: Transports molecules across membranes.
CPA4	264.17	2.12	0.24	3.62E-20	4.20E-17	Carboxypeptidase A4: Processes protein precursors and zymogens.
RELB	170.73	2.05	0.23	3.77E-20	4.20E-17	RELB Proto-Oncogene, NF-kB: Regulates immune and inflammatory responses.
PPP1R15A	646.58	1.20	0.13	3.81E-20	4.20E-17	Protein Phosphatase 1 Regulatory 15A: Modulates protein phosphatase 1 activity under stress.
TNFRSF12A	1639.28	1.00	0.11	3.89E-20	4.20E-17	TNF Receptor Superfamily Member 12A: Regulates apoptosis and inflammation.
IFIT2	153.75	2.16	0.25	9.66E-20	9.69E-17	Interferon Induced Protein With Tetratricopeptide 2: Involved in antiviral response.
TACSTD2	266.34	2.08	0.24	1.69E-19	1.58E-16	Tumor-Associated Calcium Signal Transducer 2: Mediates cell adhesion and signaling.
LTN1	861.36	1.02	0.12	8.11E-19	7.12E-16	Listerin E3 Ubiquitin Protein Ligase 1: Functions in protein quality control.
MAFF	332.44	1.77	0.21	2.84E-18	2.35E-15	MAF BZIP Transcription Factor F: Regulates oxidative stress response.
SNHG32	1788.59	0.87	0.11	1.00E-17	7.64E-15	Small Nucleolar RNA Host Gene 32: Encodes a lncRNA regulating gene expression.
GEM	555.86	1.13	0.14	1.03E-17	7.64E-15	GTP Binding Protein Overexpressed In Skeletal Muscle: Regulates signaling and cytoskeletal organization.
EPHA2	2888.19	0.89	0.11	1.12E-17	7.90E-15	EPH Receptor A2: Mediates cell migration and developmental processes.
NT5E	1692.49	0.90	0.11	4.90E-17	3.28E-14	5'-Nucleotidase Ecto: Converts nucleotides to nucleosides in purine metabolism.
MT1E	1162.49	-1.22	0.15	6.81E-17	4.35E-14	Metallothionein 1E: Binds heavy metals for detoxification.
ENC1	802.71	-1.21	0.15	2.46E-16	1.49E-13	Ectodermal-Neural Cortex 1: Regulates cytoskeletal & neuronal differentiation.
PLAUR	825.57	1.56	0.20	2.54E-16	1.49E-13	Plasminogen Activator, Urokinase Receptor: Modulates fibrinolysis and cell migration.

JUN	457.17	1.30	0.17	5.71E-16	3.21E-13	Jun Proto-Oncogene, AP-1 Transcription Factor: Regulates proliferation & differentiation.
GADD45B	317.53	1.42	0.19	9.31E-16	5.03E-13	Growth Arrest And DNA Damage Inducible Beta: Responds to stress & DNA damage.
HERPUD1	988.64	0.92	0.12	2.56E-15	1.33E-12	Homocysteine Inducible ER Protein With Ubiquitin Like Domain 1: Involved in ER stress and protein degradation.
ID3	848.64	-1.03	0.14	5.38E-15	2.70E-12	Inhibitor Of DNA Binding 3: Acts as a dominant-negative transcription regulator.
SOX4	2103.19	-0.78	0.11	1.08E-14	5.23E-12	SRY-Box Transcription Factor 4: Regulates embryonic development and cell fate.
SRXN1	2236.39	0.74	0.10	1.68E-14	7.89E-12	Sulfiredoxin 1: Reduces hyperoxidized peroxiredoxins in oxidative stress response.
CSRNP1	348.23	1.15	0.16	3.45E-14	1.56E-11	Cysteine And Serine Rich Nuclear Protein 1: Involved in apoptosis and transcription.
CYP24A1	1682.61	-0.90	0.13	7.01E-14	3.08E-11	Cytochrome P450 24A Member 1: Degrades active vitamin D metabolites.
RNF141	4367.34	-0.65	0.09	1.23E-13	5.25E-11	Ring Finger Protein 141: Functions as an E3 ubiquitin ligase.
STMN1	4187.96	-0.73	0.10	1.48E-13	6.13E-11	Stathmin 1: Regulates microtubule dynamics.
LAMB3	1112.82	1.43	0.21	1.63E-13	6.55E-11	Laminin Subunit Beta 3: Supports cell adhesion and migration.
PTPRH	314.54	1.33	0.19	2.04E-13	7.95E-11	Protein Tyrosine Phosphatase Receptor H: Modulates cell growth & differentiation.
BDNF	1245.01	-0.94	0.14	2.42E-13	9.17E-11	Brain-Derived Neurotrophic Factor: Promotes neuronal survival and synaptic growth.
TRIB1	5257.70	-0.64	0.09	3.07E-13	1.13E-10	Tribbles Pseudokinase 1: Regulates proliferation, differentiation, and survival.
NFKB2	1091.48	1.05	0.15	3.52E-13	1.27E-10	Nuclear Factor Kappa B Subunit 2: Regulates immune response and inflammation.
SFN	1513.45	0.84	0.12	4.07E-13	1.43E-10	Stratifin: Controls cell cycle and apoptosis.
RIOK3	1529.30	1.09	0.16	7.82E-13	2.68E-10	RIO Kinase 3: Functions in ribosome biogenesis and stress response.
VGF	187.25	1.47	0.22	1.08E-12	3.63E-10	VGF Nerve Growth Factor Inducible: Regulates energy homeostasis & synaptic activity.
IFRD1	836.98	0.81	0.12	1.69E-12	5.51E-10	Interferon Related Developmental Regulator 1: Modulates myogenesis and interferon response.
ZNF704	1453.92	-0.84	0.13	2.26E-12	7.21E-10	Zinc Finger Protein 704: Functions in transcriptional regulation.
SDC4	2003.87	1.03	0.16	2.58E-12	8.06E-10	Syndecan 4: Mediates cell adhesion and signaling.
PHLDB2	6948.07	-0.69	0.11	3.60E-12	1.10E-09	Pleckstrin Homology Like Domain Family B Member 2: Regulates signaling and cytoskeletal organization.
MAN1A1	4576.56	-0.72	0.11	5.51E-12	1.65E-09	Mannosidase Alpha Class 1A1: Processes N-linked glycoproteins in the Golgi.
MANF	1191.76	0.75	0.12	6.73E-12	1.97E-09	Mesencephalic Astrocyte Derived Neurotrophic Factor: Supports dopaminergic neuron survival.
LOC105376938	334.62	1.12	0.17	7.24E-12	2.08E-09	Uncharacterized LOC105376938: Predicted to be involved in cellular processes.
ZMYM5	457.50	0.94	0.15	9.02E-12	2.51E-09	Zinc Finger MYM-Type Containing 5: Regulates transcription.
MXD1	188.10	1.81	0.28	9.09E-12	2.51E-09	MAX Dimerization Protein 1: Acts as a transcriptional repressor.

TXNRD1	10579.12	0.68	0.11	9.67E-12	2.61E-09	Thioredoxin Reductase 1: Modulates oxidative stress and redox signaling.
MAP1LC3B	779.13	0.80	0.13	1.40E-11	3.71E-09	Microtubule Associated Protein 1 Light Chain 3 Beta: Involved in autophagy.
PARD6B	1304.70	0.71	0.11	1.45E-11	3.78E-09	Par-6 Family Cell Polarity Regulator Beta: Establishes cell polarity.
HYOU1	4093.57	0.63	0.10	1.69E-11	4.32E-09	Hypoxia Up-Regulated 1: Regulates cellular hypoxia response.
GPAT3	968.10	0.74	0.12	2.17E-11	5.44E-09	Glycerol-3-Phosphate Acyltransferase 3: Catalyzes triglyceride biosynthesis.
CYP4F11	374.36	1.35	0.22	3.94E-11	9.72E-09	Cytochrome P450 Family 4 Subfamily F11: Metabolizes drugs, cholesterol, and lipids.
LPIN1	727.29	1.08	0.18	4.85E-11	1.18E-08	Lipin 1: Regulates triglyceride and phospholipid biosynthesis.
LURAP1L	106.36	1.73	0.29	7.33E-11	1.75E-08	Leucine-Rich Adaptor Protein 1-Like: Potential role in cell signaling and regulation.
GAS5	3004.72	0.84	0.14	8.95E-11	2.10E-08	Growth Arrest Specific 5: Regulates cell growth, apoptosis, & growth arrest response.
TIPARP	2338.37	0.79	0.13	9.42E-11	2.17E-08	TCDD-Inducible Poly(ADP-Ribose) Polymerase: Involved in DNA repair, transcription, and stress response.
PDE4B	2279.61	-0.91	0.15	1.10E-10	2.49E-08	Phosphodiesterase 4B: Hydrolyzes cAMP, regulating signaling & inflammatory responses.
HSP90B1	20855.42	0.71	0.12	1.38E-10	3.08E-08	Heat Shock Protein 90 Beta1: Chaperone for protein folding, stabilization, & degradation.
DUSP6	2571.55	-0.86	0.14	1.43E-10	3.13E-08	Dual Specificity Phosphatase 6: Regulates MAP kinases, influencing cell proliferation and differentiation.
SYNE2	3958.58	-0.59	0.10	1.88E-10	4.07E-08	Spectrin Repeat Containing Nuclear Envelope Protein 2: Structural nuclear envelope protein for positioning and anchorage.
ETV1	522.95	-0.91	0.15	2.34E-10	4.99E-08	Ets Variant Transcription Factor 1: Regulates genes linked to proliferation, differentiation, and apoptosis.
CHML	849.44	-0.82	0.14	2.61E-10	5.43E-08	CHM Like, Rab Escort Protein: Supports Rab GTPase prenylation for vesicle trafficking.
CBX5	3717.74	-0.71	0.12	2.63E-10	5.43E-08	Chromobox 5: Heterochromatin component, maintaining chromatin structure and function.
CYB5B	13958.09	-0.52	0.09	2.83E-10	5.76E-08	Cytochrome B5: Electron transporter in fatty acid desaturation and elongation.
HMOX1	282.05	1.07	0.18	3.22E-10	6.46E-08	Heme Oxygenase 1: Degrades heme, providing cytoprotective & anti-inflammatory effects.
UPP1	174.96	1.53	0.26	3.51E-10	6.95E-08	Uridine Phosphorylase 1: Catalyzes uridine phosphorolysis in the pyrimidine salvage pathway.
AREG	6584.40	0.94	0.16	3.65E-10	7.11E-08	Amphiregulin: Growth factor binding EGFR, promoting proliferation & differentiation.
KRT15	330.60	1.48	0.26	4.56E-10	8.78E-08	Keratin 15: Structural keratin protein in epithelial cells.
CD55	987.18	1.19	0.21	4.75E-10	9.02E-08	CD55: Decay-accelerating factor, protecting cells from complement-mediated lysis.

UBC	22953.39	0.52	0.09	6.58E-10	1.23E-07	Ubiquitin C: Encodes ubiquitin for protein degradation via the proteasome system.
OASL	117.45	1.51	0.27	6.80E-10	1.26E-07	2'-5'-Oligoadenylate Synthetase-Like: Interferon-stimulated antiviral immune response enzyme.
LOC730268	109.31	1.59	0.28	8.43E-10	1.54E-07	Uncharacterized LOC730268: Predicted gene with unknown function.
LAMC2	872.59	0.99	0.18	9.00E-10	1.62E-07	Laminin Subunit Gamma 2, Component of laminin-5: aiding adhesion, migration, and differentiation.
FAM111A	780.42	-1.00	0.18	9.65E-10	1.72E-07	Family With Sequence Similarity 111 A: Involved in DNA replication, repair, and Kenny-Caffey syndrome.
CRELD1	254.14	1.16	0.21	1.09E-09	1.91E-07	Cysteine-Rich with EGF-Like Domains 1: Regulates heart development and congenital defects.
TFE3	229.32	1.06	0.19	1.13E-09	1.96E-07	Transcription Factor Binding to IGHM Enhancer 3: Regulates growth, proliferation, and differentiation; cancer-associated.
LOC101927751	359.07	0.93	0.17	1.19E-09	2.04E-07	Uncharacterized LOC101927751: Predicted gene with unknown function.
MKX	724.85	-0.75	0.13	1.28E-09	2.16E-07	Mohawk Homeobox: Transcription factor for tendon development and maintenance.
DNAJC27	182.63	1.24	0.22	1.33E-09	2.22E-07	DnaJ Heat Shock Protein Family (Hsp40) Member C27: Co-chaperone regulating small GTPases in signaling.
HMGCS1	2819.63	0.92	0.17	1.76E-09	2.91E-07	3-Hydroxy-3-Methylglutaryl-CoA Synthase 1: Key enzyme in cholesterol and isoprenoid biosynthesis.
TMEM225B	90.50	1.76	0.32	1.82E-09	2.98E-07	Transmembrane Protein 225B: involved in membrane processes.
ITPKC	312.03	0.96	0.17	2.03E-09	3.28E-07	Inositol-Trisphosphate 3-Kinase C: Enzyme in calcium signaling via inositol trisphosphate phosphorylation.
SLC25A25	605.95	0.79	0.14	2.15E-09	3.43E-07	Solute Carrier 25A25: Mitochondrial carrier transporting adenine nucleotides.
HBEGF	208.35	1.07	0.20	2.59E-09	4.09E-07	Heparin-Binding EGF-Like Growth Factor: EGFR-binding growth factor for healing, proliferation, and differentiation.
LDLR	2647.74	0.72	0.13	2.67E-09	4.17E-07	Low Density Lipoprotein Receptor: Internalizes LDL, regulating cholesterol homeostasis.
DDR2	107.63	1.51	0.28	3.23E-09	4.98E-07	Discoidin Domain Receptor Tyrosine Kinase 2: Regulates adhesion, proliferation, and extracellular matrix remodeling.

Genes significantly associated with vitamin D levels as identified from RNA-sequencing of the overexpression *SDR42E1* HCT116 model (FDR < 5E−07). Abbreviations: FDR, P-value adjusted using Benjamini-Hochberg in DESeq2.

Supplementary Figures

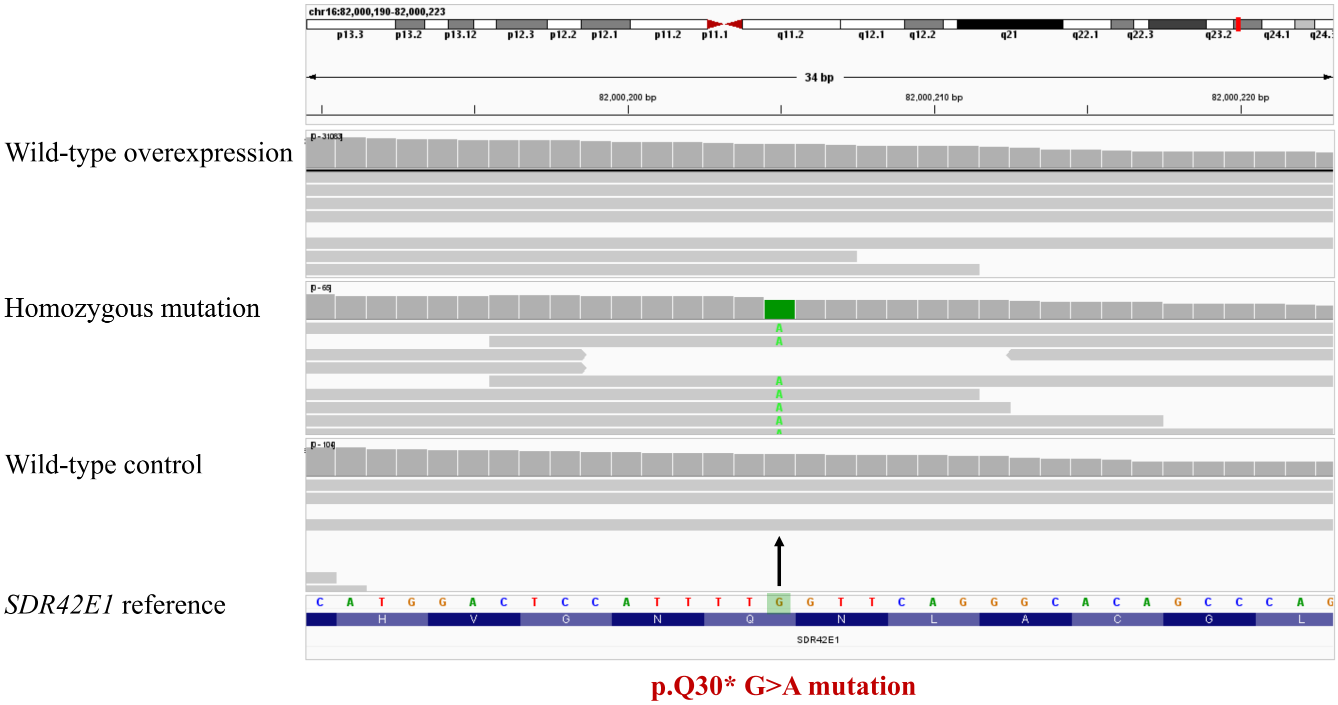

Figure S1 Validation of SDR42E1 gene knock-in and overexpression in genome-edited HCT116 cells.

Integrative Genomics Viewer (IGV) of bam files analysis included the wild-type overexpression of SDR42E1, homozygous mutation of SDR42E1, and a control from unedited HCT116 wild-type cells. The CRISPR/Cas9 editing resulted in a homozygous base substitution of a guanine to an adenine at position 30 (p.Q30* G>A). This corresponds to a reverse orientation mutation (p.Q30* C>T) at position 82000205 of chromosome 16, introducing a premature stop codon. Among the 19,713 total reads at this position, 19,696 reads showed the reference allele (G) and 5 reads showed the alternative allele (A) in the overexpressed cells. In the SDR42E1 homozygous mutated cells, all 34 reads displayed the alternative allele (A), while the control cells exhibited all 61 reads with the reference allele (G).

frontiers

A

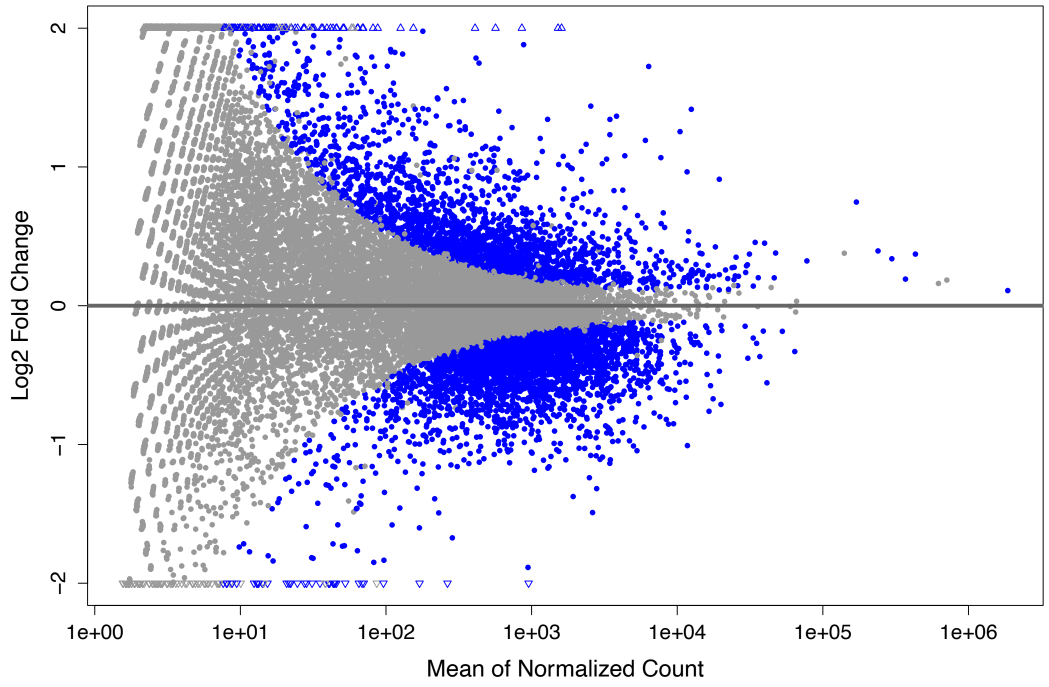

B

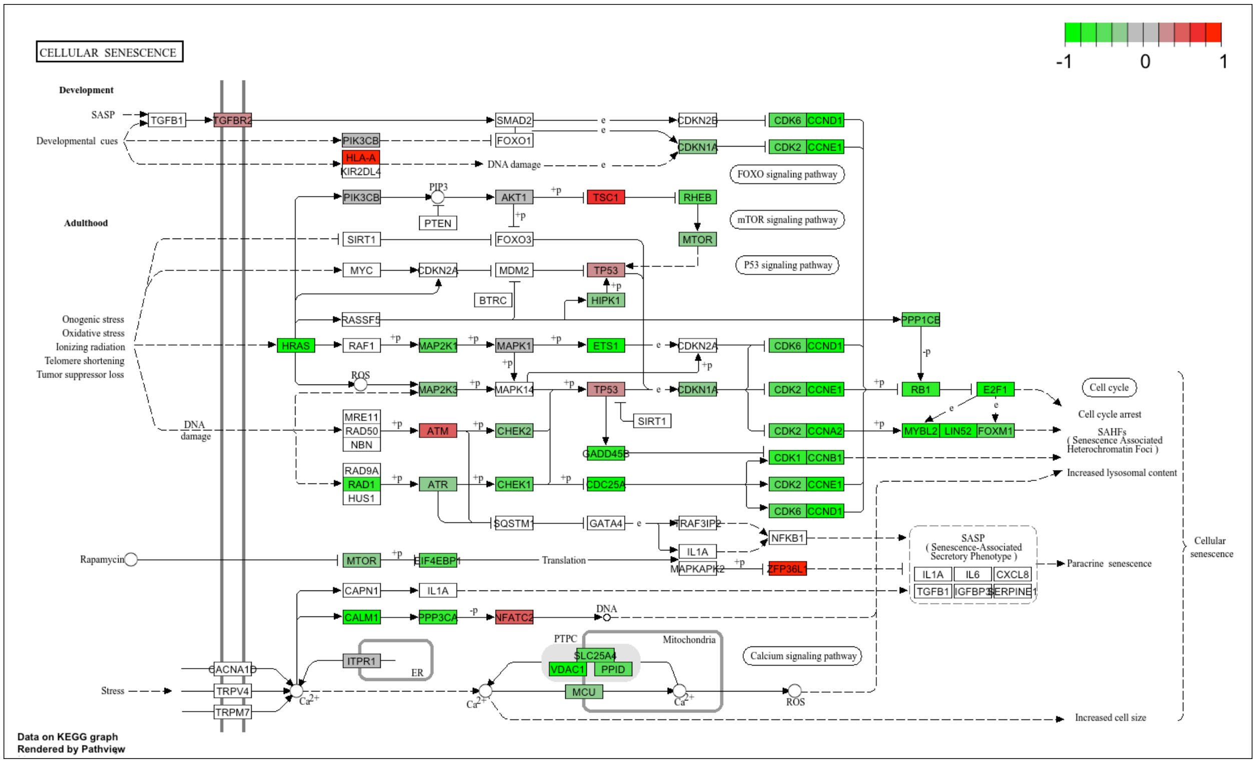

C

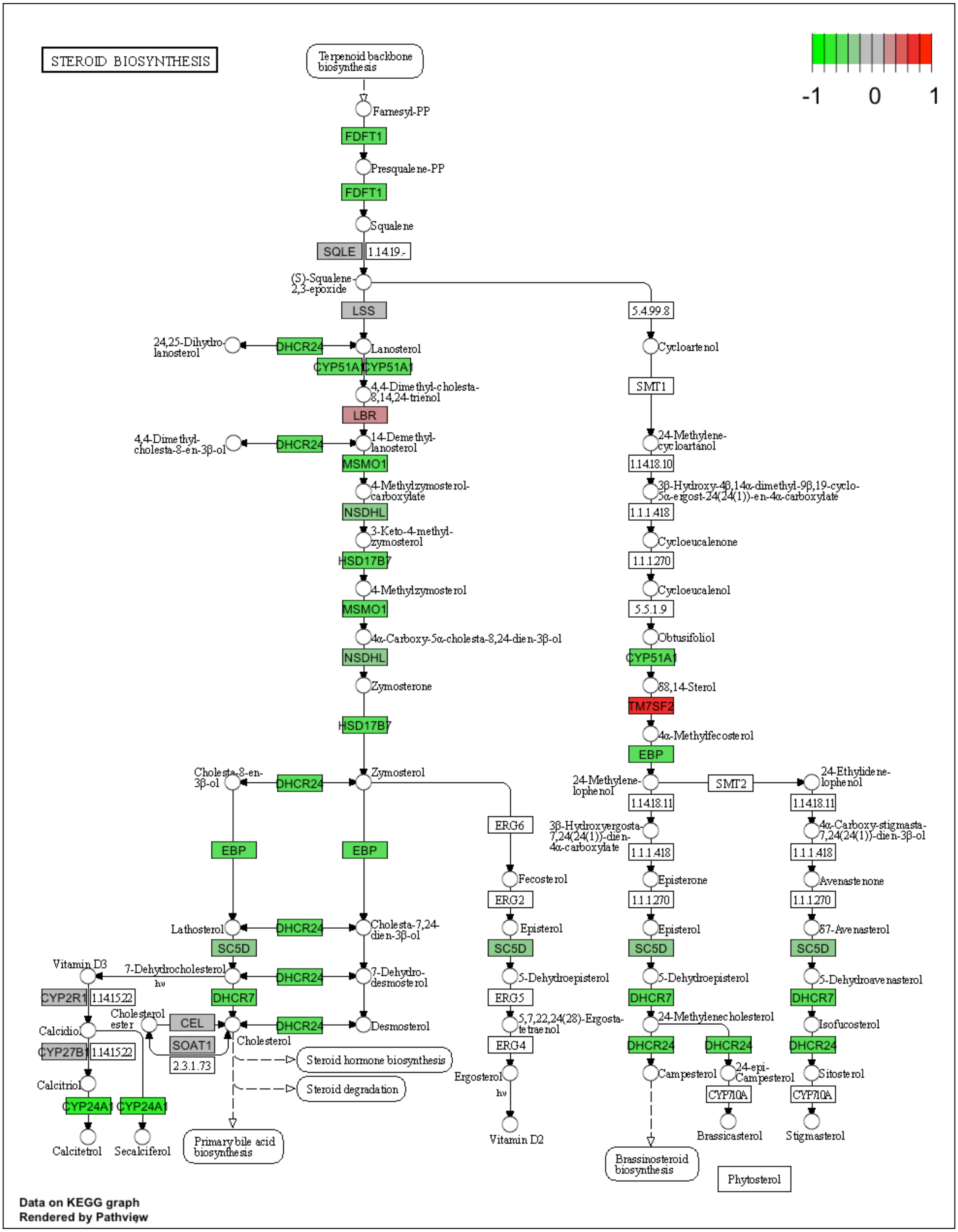

STEROID BIOSYNTHESIS
Terpenoid backbone biosynthesis
-1
0
1
Farnesyl-PP
FDFT1
Presqualene-PP
FDFT1
Squalene
SQLE
1.14.19.-
(S)-Squalene-2,3-epoxide
LSS
5.4.99.8
24,25-Dihydro-lanosterol
DHCR24
Lanosterol
Cycloartenol
CYP51A1
CYP51A1
4,4-Dimethyl-cholesta-8,14,24-trienol
SMT1
LBR
4,4-Dimethyl-cholesta-8-en-3β-ol
DHCR24
14-Demethyl-lanosterol
24-Methylene-cycloartanol
MSMO1
1.14.18.10
4-Methylzymosterol-carboxylate
3β-Hydroxy-4β,14α-dimethyl-9β,19-cyclo-5α-ergost-24(24(1))-en-4α-carboxylate
NSDHL
1.1.1.418
3-Keto-4-methyl-zymosterol
Cycloeucalenone
HSD17B7
1.1.1.270
4-Methylzymosterol
Cycloeucalenol
MSMO1
5.5.1.9
4α-Carboxy-5α-cholesta-8,24-dien-3β-ol
Obtusifoliol
NSDHL
CYP51A1
Zymosterone
δ8,14-Sterol
TM7SF2
HSD17B7
4α-Methylfecosterol
EBP
Cholesta-8-en-3β-ol
DHCR24
Zymosterol
24-Methylene-lophenol
SMT2
24-Ethylidene-lophenol
1.14.18.11
1.14.18.11
ERG6
3β-Hydroxyergosta-7,24(24(1))-dien-4α-carboxylate
4α-Carboxy-stigmasta-7,24(24(1))-dien-3β-ol
EBP
EBP
1.1.1.418
1.1.1.418
Fecosterol
Episterone
Avenastenone
ERG2
1.1.1.270
1.1.1.270
Lathosterol
DHCR24
Cholesta-7,24-dien-3β-ol
Episterol
Episterol
δ7-Avenasterol
SC5D
SC5D
SC5D
SC5D
Vitamin D3
7-Dehydrocholesterol
hν
DHCR24
7-Dehydro-desmosterol
5-Dehydroepisterol
5-Dehydroepisterol
5-Dehydroavenasterol
CYP2R1
1.14.15.22
DHCR7
ERG5
DHCR7
DHCR7
Cholesterol ester
CEL
DHCR24
Desmosterol
5,7,22,24(28)-Ergosta-tetraenol
24-Methylenecholesterol
Isofucosterol
Calcidiol
SOAT1
Cholesterol
ERG4
DHCR24
DHCR24
DHCR24
CYP27B1
1.14.15.22
2.3.1.73
Steroid hormone biosynthesis
Ergosterol
24-epi-Campesterol
Campesterol
Sitosterol
Calcitriol
Steroid degradation
hν
CYP710A
CYP710A
CYP24A1
CYP24A1
Calcitetrol
Secalciferol
Primary bile acid biosynthesis
Vitamin D2
Brassicasterol
Stigmasterol
Brassinosteroid biosynthesis
Phytosterol
Data on KEGG graph
Rendered by Pathview

D

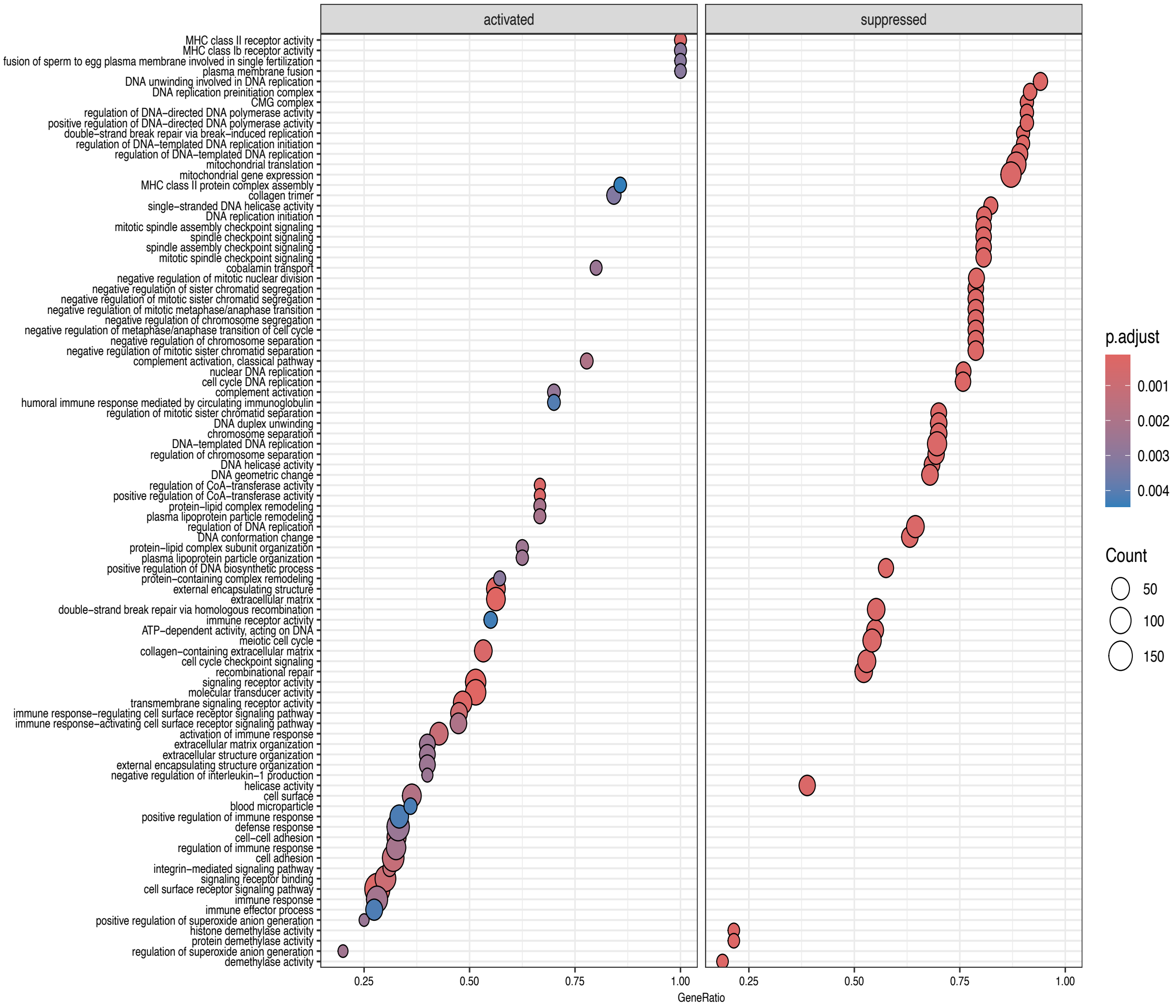

Figure S2 Extensive alterations in genes and pathways in the SDR42E1 knock-in model.

(A) The MDS plot depicts the correlation between log_2 fold change and the mean of normalized counts in SDR42E1 knock-in, highlighting significant DEGs in blue (adjusted P-value < 0.05). KEGG pathway diagrams depict the expression profiles of genes implicated in **(B)** cellular senescence, and in **(C)** steroid hormone biosynthesis by the SDR42E1 knock-in, created with the R/Pathview package. Genes upregulated are highlighted in red, while those downregulated are marked in green. **(D)** The dot plot shows enriched GSE pathways for DEGs. The Y-axis represents the GSE pathways, and the X-axis represents the ratio of genes enriched in the GSE pathway. A dot plot categorizes enriched KEGG pathways for DEG.

A

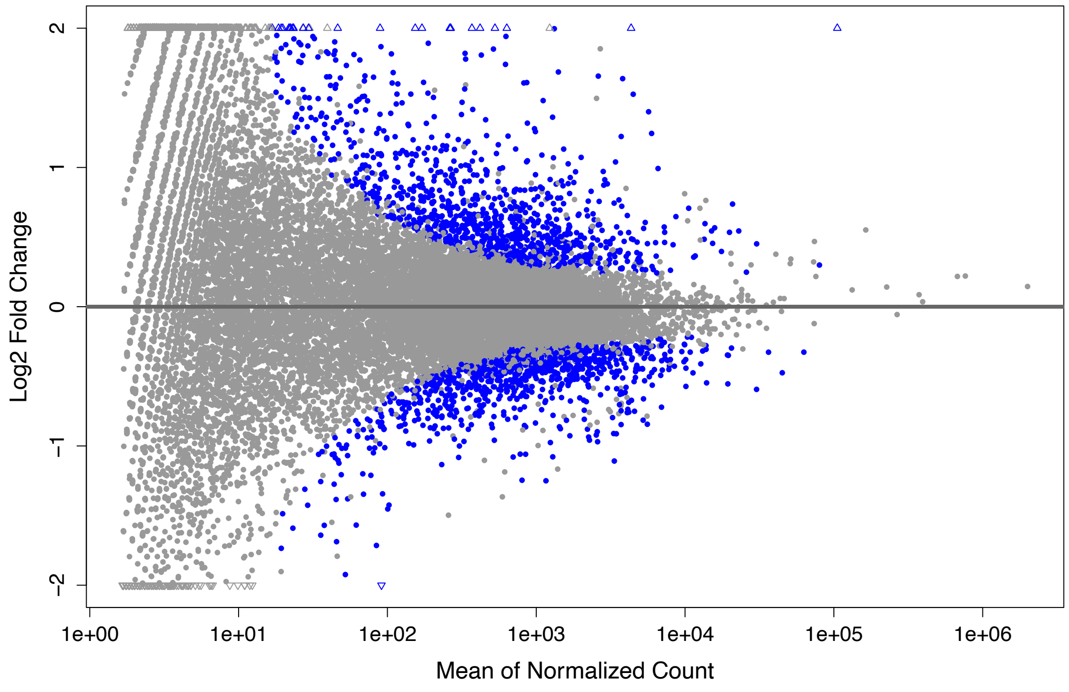
Log2 Fold Change
Mean of Normalized Count
1e+00
1e+01
1e+02
1e+03
1e+04
1e+05
1e+06
2
1
0
−1
−2

B

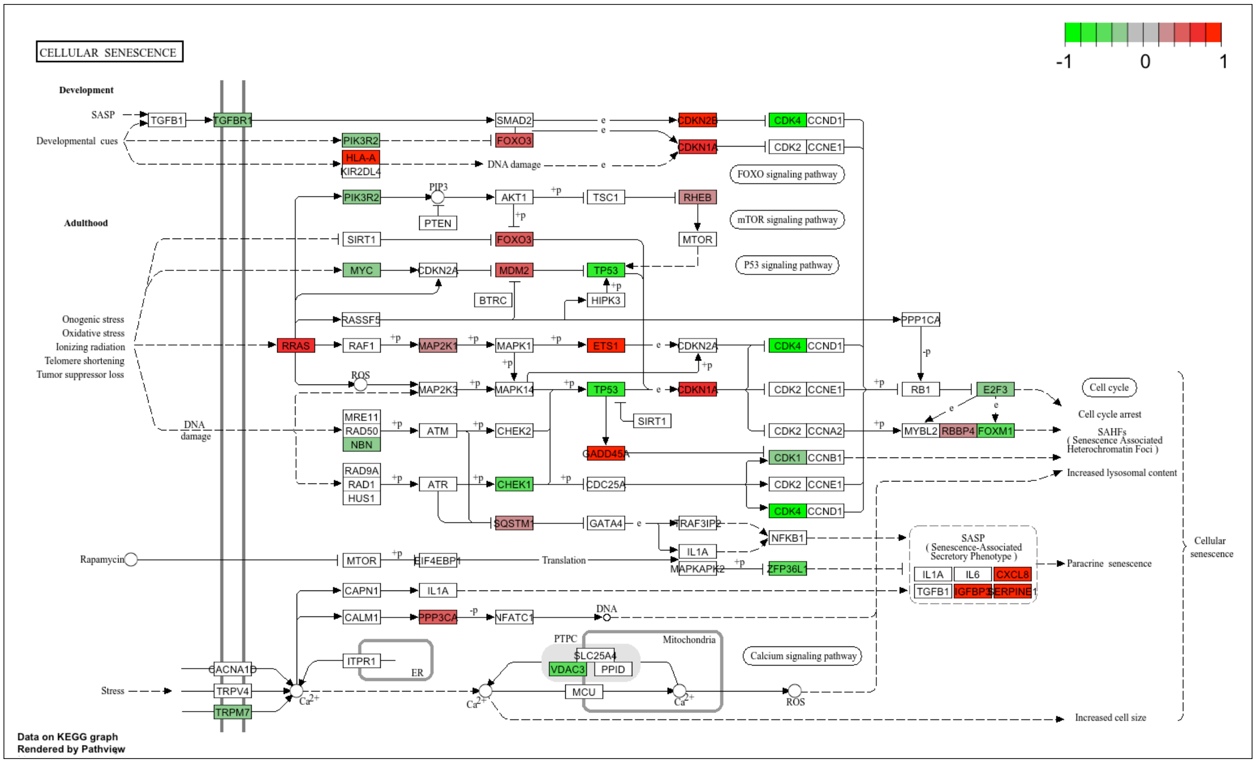
CELLULAR SENESCENCE
-1
0
1
Development
SASP
Developmental cues
Adulthood
Oncogenic stress
Oxidative stress
Ionizing radiation
Telomere shortening
Tumor suppressor loss
DNA damage
Rapamycin
Stress
TGFB1
TGFBR1
PIK3R2
HLA-A
KIR2DL4
SMAD2
FOXO3
CDKN2B
CDKN1A
CDK4
CCND1
CDK2
CCNE1
FOXO signaling pathway
mTOR signaling pathway
P53 signaling pathway
PIP3
AKT1
TSC1
RHEB
PTEN
MTOR
SIRT1
MYC
CDKN2A
MDM2
TP53
BTRC
HIPK3
RASSF5
PPP1CA
RRAS
RAF1
MAP2K1
MAPK1
ETS1
ROS
MAP2K3
MAPK14
RB1
E2F3
MRE11
RAD50
NBN
ATM
CHEK2
MYBL2
RBBP4
FOXM1
GADD45A
CDK1
CCNB1
CCNA2
RAD9A
RAD1
HUS1
ATR
CHEK1
CDC25A
SQSTM1
GATA4
RAF3IP2
IL1A
NFKB1
MAPKAPK2
ZFP36L1
EIF4EBP1
Translation
CAPN1
CALM1
PPP3CA
NFATC1
DNA
ITPR1
ER
PTPC
Mitochondria
SLC25A4
VDAC3
PPID
MCU
CACNA1D
TRPV4
TRPM7
Calcium signaling pathway
ROS
Cell cycle
Cell cycle arrest
SAHFs
(Senescence Associated Heterochromatin Foci)
Increased lysosomal content
SASP
(Senescence-Associated Secretory Phenotype)
IL6
CXCL8
IGFBP3
SERPINE1
Paracrine senescence
Cellular senescence
Increased cell size
Data on KEGG graph
Rendered by Pathview

C

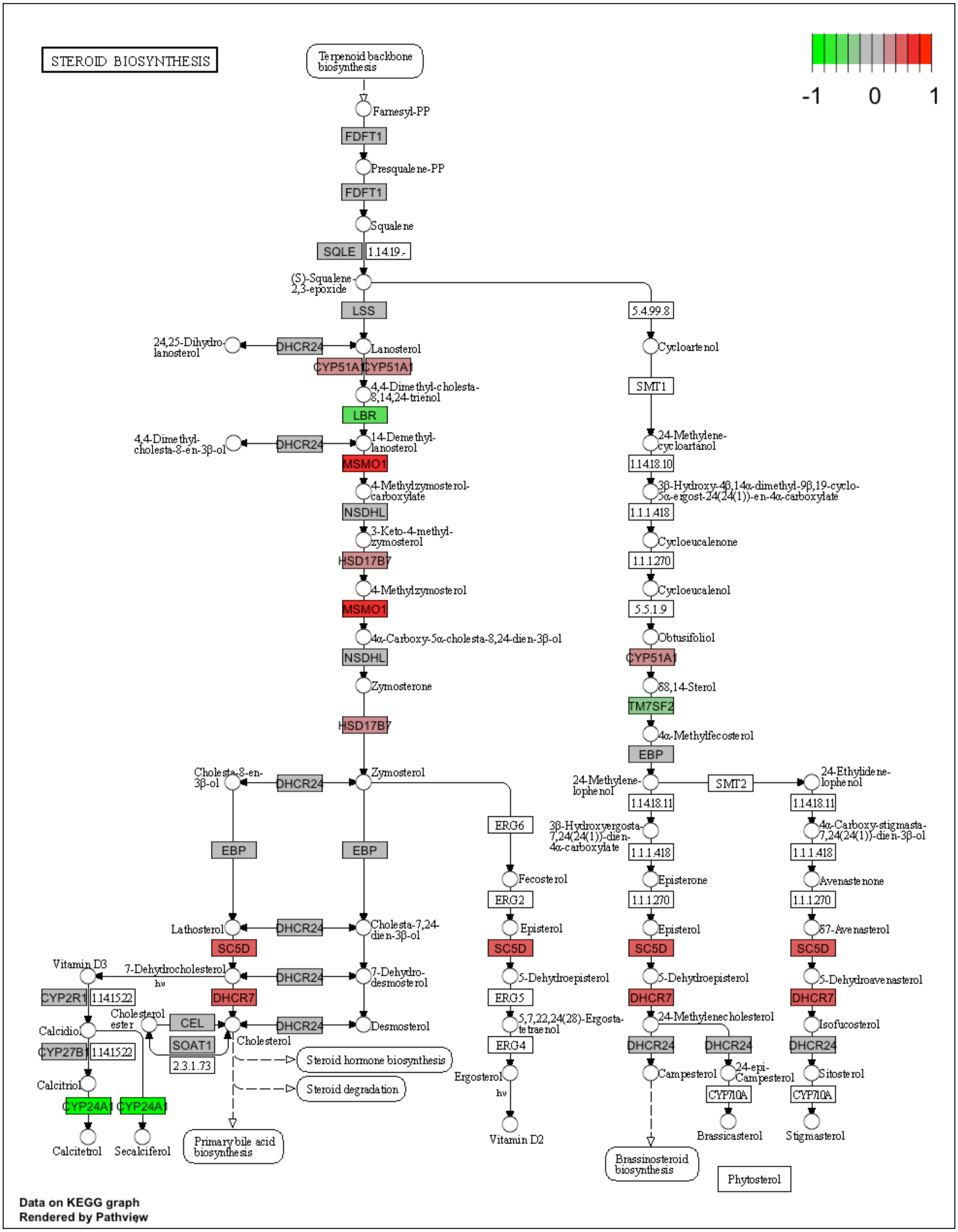
STEROID BIOSYNTHESIS
Terpenoid backbone biosynthesis
-1
0
1
Farnesyl-PP
FDFT1
Presqualene-PP
FDFT1
Squalene
SQLE
1.14.19.-
(S)-Squalene-2,3-epoxide
LSS
5.4.99.8
24,25-Dihydro-lanosterol
DHCR24
Lanosterol
CYP51A1
CYP51A1
Cycloartenol
4,4-Dimethyl-cholesta-8,14,24-trienol
SMT1
LBR
4,4-Dimethyl-cholesta-8-en-3β-ol
DHCR24
14-Demethyl-lanosterol
24-Methylene-cycloartanol
MSMO1
1.14.18.10
4-Methylzymosterol-carboxylate
3β-Hydroxy-4β,14α-dimethyl-9β,19-cyclo-5α-ergost-24(24(1))-en-4α-carboxylate
NSDHL
1.1.1.418
3-Keto-4-methyl-zymosterol
Cycloeucalenone
HSD17B7
1.1.1.270
4-Methylzymosterol
Cycloeucalenol
MSMO1
5.5.1.9
4α-Carboxy-5α-cholesta-8,24-dien-3β-ol
Obtusifoliol
NSDHL
CYP51A1
Zymosterone
δ8,14-Sterol
TM7SF2
HSD17B7
4α-Methylfecosterol
EBP
Cholesta-8-en-3β-ol
DHCR24
Zymosterol
24-Methylene-lophenol
SMT2
24-Ethylidene-lophenol
1.14.18.11
1.14.18.11
ERG6
3β-Hydroxyergosta-7,24(24(1))-dien-4α-carboxylate
4α-Carboxy-stigmasta-7,24(24(1))-dien-3β-ol
EBP
EBP
1.1.1.418
1.1.1.418
Fecosterol
Episterone
Avenastenone
ERG2
1.1.1.270
1.1.1.270
Lathosterol
DHCR24
Cholesta-7,24-dien-3β-ol
Episterol
Episterol
δ7-Avenasterol
SC5D
SC5D
SC5D
SC5D
Vitamin D3
7-Dehydrocholesterol
DHCR24
7-Dehydro-desmosterol
5-Dehydroepisterol
5-Dehydroepisterol
5-Dehydroavenasterol
hν
CYP2R1
1.14.15.22
DHCR7
ERG5
DHCR7
DHCR7
Cholesterol ester
CEL
DHCR24
Desmosterol
5,7,22,24(28)-Ergosta-tetraenol
24-Methylenecholesterol
Isofucosterol
Calcidiol
SOAT1
Cholesterol
ERG4
DHCR24
DHCR24
DHCR24
CYP27B1
1.14.15.22
2.3.1.73
Steroid hormone biosynthesis
Ergosterol
Campesterol
24-epi-Campesterol
Sitosterol
hν
Calcitriol
Steroid degradation
CYP710A
CYP710A
CYP24A1
CYP24A1
Calcitetrol
Secalciferol
Primary bile acid biosynthesis
Vitamin D2
Brassicasterol
Stigmasterol
Brassinosteroid biosynthesis
Phytosterol
Data on KEGG graph
Rendered by Pathview

D

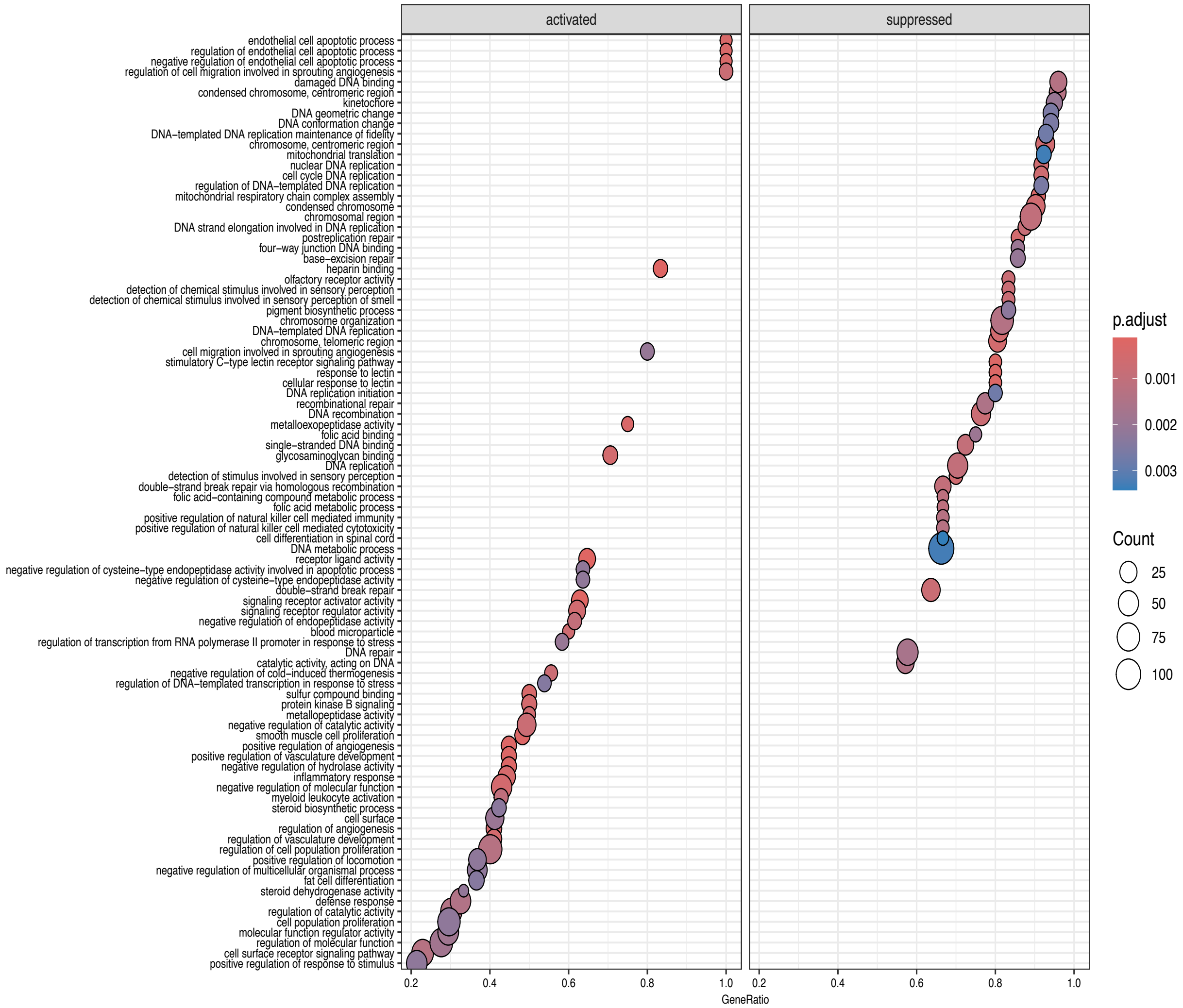

Figure S3 Significant changes in genes and pathways in the SDR42E1 wild-type overexpression model.

(A) The MDS plot exhibits the correlation between $\log_2$ fold change and the mean of normalized counts in SDR42E1 overexpression, highlighting significant DEGs in blue (adjusted P-value < 0.05). A dot plot categorizes enriched KEGG pathways for DEG. KEGG pathway diagrams depict the expression profiles of genes implicated in **(B)** cellular senescence, and in **(C)** steroid hormone biosynthesis by the SDR42E1 wild-type overexpression created with the R/Pathview package. Genes upregulated are highlighted in red, while those downregulated are marked in green. **(D)** Dot plot displays enriched GSE pathways for DEGs. The Y-axis represents the GSE pathways, and the X-axis represents the ratio of genes enriched in the GSE pathway.
